# The balance evaluation systems test (BESTest), mini-BESTest and brief-BESTest as clinical tools to assess balance control across different populations: A reliability generalization meta-analysis

**DOI:** 10.1371/journal.pone.0318302

**Published:** 2025-04-03

**Authors:** Ana-Belén Meseguer-Henarejos, Juan-José López-García, José-Antonio López-Pina, Ignacio Martínez-González-Moro, Ángel Martínez-Carrasco

**Affiliations:** 1 Department of Physiotherapy, University of Murcia, Murcia, Spain; 2 Department of Basic Psychology and Methodology, University of Murcia, Murcia, Spain; CHU Nantes: Centre Hospitalier Universitaire de Nantes - Nantes Université, FRANCE

## Abstract

**Background:**

The Balance Evaluation Systems Test (BESTest) and two abbreviated versions, Mini-BESTest and Brief-BESTest are used to assess functioning of balance control systems. Its reliability across different populations remains to be determined.

**Objective:**

The present study followed reliability generalization procedures to estimate an average internal consistency and inter and intra-rater reliability for the BESTest, Mini-BESTest and Brief-BEStest. In this study, the heterogeneity of reliability coefficients in each instrument is evaluated. If heterogeneity is significant, a moderator analysis is performed to identify the characteristic which explains such variability.

**Methods:**

A search of the PubMed, Embase, PsycINFO, Web of Science, Scopus and CINAHL databases was carried out to February 10^th^ 2024. Two reviewers independently selected empirical studies published in English or Spanish that applied the BESTest, Mini-BESTest and/or Brief-BESTest and reported any reliability coefficient and/or internal consistency with data at hand.

**Results:**

Sixty-four studies reported any reliability estimate BESTest, Mini-BESTest and/or Brief-BESTest scores (N. = 5225 participants). Mean Cronbach alpha for the Mini-BESTest and Brief-BESTest (total score = 0.92) indicating no variability in estimated internal consistency. Likewise, no variability was obtained for inter-rater and intra-rater mean agreement of the BESTest (ICC = 0.97; 0.94), Mini-BESTest (ICC = 0.95; 0.94) and Brief-BESTest (ICC = 0.96; 0.95). Mean scores, standard deviation of scores, mean age, gender, population type, mean history of the disorder, disease, raters´ experience, number of raters, rater formation, continent of study and design type presented statistically significant relationships with ICC and/or Cronbach´s alpha for BESTest and the two abbreviated versions.

**Conclusions:**

The mean intraclass correlations and Cronbach alpha obtained for BESTest, Mini-BESTest and Brief-BESTest exhibited an excellent inter and intra-rater reliability and internal consistency. The average reliability obtained three scales adequate to be applied for screening balance problems in different populations. Some continuous and categorical moderator variables increase reliability and internal consistency of these scales.

## Introduction

The Balance Evaluation Systems Test (BESTest) and two abbreviated versions, Mini-BESTest and Brief-BESTest are used to assess functioning of balance control systems [1]. Balance control is quite complex and results from a set of interacting systems [2–7]. Six underlying balance systems contribute to balance control using a systems model of motor control as the theoretical framework [1]: biomechanical constraint, stability limits/verticality, anticipatory postural adjustments, postural responses, sensory orientation and gait stability. An impairment in one or more of these systems leads to postural instability or balance problems.

Balance impairments or problems can be present in patients with a medical condition such as stroke, Parkinson´s disease, multiple sclerosis, spinal cord injury, cervical spondylotic myelopathy, myotonic dystrophy type 1, spinocerebellar ataxia, femoral or vertebral fracture, type 2 diabetes, total knee arthroplasty, cancer, end-stage disease, or chronic obstructive pulmonary disease, as well as in older adults, people with increased risk of falling and school-aged children. Impairments or deficits in balance control lead to limitations in daily life activities, reduced ambulatory capacity, limitation in social participation, affect life quality, and increased risk of falls [8–11].

These scales are applied manually to determine whether the patient has balance problems and assess their cause, unlike other outcome measures, which only reveal the existence of an equilibrium problem such as One Leg Stand, Functional Reach Test and Timed Up and Go [12]. The BESTest, development by Horak et al. [1], contains 36 items to assess balance impairments in 6 categories or systems previously indicated. Each item was scored on a 0-to-3-point scale, with a higher score indicating better balance. Its administration takes a considerable amount of time (20–30 minutes), which may not be feasible and practical for routine clinical use. Thus, two abbreviated versions of the BESTest take approximately half of the time to be administrated (10–15 minutes) for Mini-BESTest and 7–10 minutes for Brief-BESTest [13]. The Mini-BESTest developed by Franchignoni et al. [14] consists of 14 items from 4 of 6 sections from the BESTest (sections III, IV, V and VI) but does not include the biomechanical constraints and stability limits from the six sections of the BESTest. Each item is scored on a 3 level from 0 to 2 (total score equals 28 points) [15]. The Mini-BESTest´s lack of items assessing mechanical constraints or limits of stability could inhibit its sensitivity when applied to people with musculoskeletal impairments or impaired limits of stability [16]. Brief-BESTest, developed by Padget et al. [16] assesses all sections of the BESTest using the most representative item of each section [15]. Of these three scales, that most used in observational and experimental studies is the Mini-BESTest, followed by the BESTest and the Brief-BESTest, according to the search conducted in different databases.

From their original validation in the USA, the BESTest, Mini-BESTest and Brief-BESTest have been used in many cultures and countries, such as Sweden [17,18], Thailand [19–23], Brazil [24–26], Portugal [27–28], Iran [29,30], Canada [31], Belgium [32], Greece [33], Japan [34], Norway [35], Slovenia [36], Croatia [36], Turkey [37–39], Germany [40], China [41,42], Spain [43], Saudi Arabia [44,45] and Italy [18,46–49].

In order to be efficient, a measurement tool must have good psychometric properties like reliability, measurement error verified by the Standard Error of Measurement (SEM) and/or Bland-Altman plot, validity and responsiveness. This study focused on the internal consistency and inter and intra-rater reliability (test-retest) of the BESTest, Mini-BESTest and Brief-BESTest. Reliability and internal consistency are not inherent test properties and may vary each time it is applied to a different sample of participants [50,51]. Whenever a study makes use of a scale, authors should report a reliability estimate with data available [52]. However, in experimental study’s authors often do not report reliability estimates based on their own participants’ scores, rather it is common to find references to reliability obtained in the original validation study of the test. Checking reliability of test application scores is of paramount importance in ensuring that the measurement itself is reliable and because reliability affects effect sizes. If test scores are less reliable, the effect size on these instruments can be attenuated [53]. In short, if the scale does not produce reliable scores, diagnosis might be inaccurate and effectiveness of treatments to improve or maintain balance cannot be adequately tested.

Nevertheless, a representative reliability value of an instrument can be obtained by integrating the various reliability estimates obtained in studies using meta-analytic methods. This is often referred to as reliability generalization (RG) [54]. Additionally, if heterogeneity exists between reliability estimates based on the same test, an RG meta-analysis enables us to examine whether some study characteristics (i.e., moderators) could explain the variability of reliability coefficients [55,56]. Examples of study characteristics which may affect reliability are mean and variability of test scores, target population, or whether the original version or an adaptation (cultures or countries) of the test has been used.

Currently, no meta-analysis has been performed to generalize the reliability of the BESTest, MiniBESTes and Brief-BESTest. The objectives of this RG study are to (i) estimate an average internal consistency and inter and intra-rater reliability for the BESTest, MiniBESTest and Brief-BEStest, and (ii) assess whether there exists large heterogeneity between reliability estimates for the same instrument and, if so, perform moderator analyses to identify study characteristics which account for such variability.

## Methods

We used the Preferred Reporting Items for Systematic Reviews and Meta-Analyses to guide the reporting of the current review [57]. The review protocol was registered at the International Prospective Register of Systematic Reviews (PROSPERO: CRD42024540512).

### Identification and selection of studies

The identification and selection of studies to conduct this reliability generalization study was carried out according to five criteria: a) empirical studies (observational and experimental), b) the sample is from patients with a clinical disorder or a normal population, c) studies had to report at least an alpha coefficient to assess internal consistency and/or an intraclass correlation coefficient to assess inter-rater and/or intra-rater/test-retest reliability, d) must be published before 10 February 2024, and e) must be published in English or Spanish. Thesis or dissertations, conference abstracts, letters to editors, study protocols, guidelines, case reports, narrative review, systematic review, meta-analysis, book chapter, qualitative study and consensus-based recommendations were excluded.

To locate studies, the following electronic databases were consulted: PubMed, Embase, PsycINFO, Web of Science, Scopus and CINAHL. Forward and backward citation tracing was used, and reference lists of studies were manually checked for additional studies. Supplementary S1 Table summarizes search strategies for all databases.

After the bibliographic search phase, in the first screening, duplicated articles were removed. After that, retrieved articles were filtered based on title and abstract. All titles and abstracts were independently screened by two blinded reviewers (ABMH, JJLG) and full-text of the potential relevant articles were analyzed in-depth to examine their eligibility. If an eligible article assessed different population samples, each sample was considered as separate sample. Disagreements were resolved by consensus, with a third assessor (JALP) consulted if necessary.

### Assessment of study characteristics

Substantive and methodological characteristics were extracted with a view to examining the influence of moderating variables on reliability estimates [57]. For BESTest, Mini-BESTest and Brief-BESTest, the following methodological characteristics were coded: scale version (original vs adapted), design type (observational vs experimental), study approach (psychometric vs applied), sample size, experience of raters (yes vs no, mean in years), interrater interval (in days), number of raters, sample size for interrater agreement, intra-rater interval (in days), number of raters, and sample size for intra-rater agreement. In the case of the Mini-BESTest, the maximum scale score (28 vs 32) was included according to two possible lengths (14 and 16 items). In addition, the following substantive variables were coded: age of sample (mean and standard deviation), reference population (adults 18–65 years, adults over 65 years, children and adolescents), country and continent where study was conducted, gender distribution (%female), target population (clinical, normal non-institutionalized population, normal institutionalized population), disease type, disease history (mean and standard deviation in years), experience of raters (physical therapist, medical doctor, other), and year of study.

### Data extraction

To assess reliability of data extraction, two assessors independently (ABMH, JALP) coded characteristics from all studies containing information from BESTest, Mini-BESTest and Brief-BESTest. If a study contained more than one sample with relevant information on reliability, separate coding was performed for each sample. Cohen’s kappa coefficients were calculated for inter-rater agreement of the categorical moderator variables, while intraclass correlations were calculated for the continuous moderator variables. Cohen’s kappa coefficient ranged from 0.883 to 1, while the intraclass correlation for continuous variables ranged from 0.569 to 1. Inconsistencies among raters were resolved by consensus.

Reliability coefficients were a source of heterogeneity as one or more alpha coefficient and/or inter-rater and/or intra-rater agreement could appear in articles. Table 1 shows number of studies, number of samples, and sample size for BESTest, Mini-BESTest y Brief-BESTest.

**Table 1:** Characteristics of the BESTest, Mini-BESTest and Brief-BESTest.

	BESTest	Mini-BESTest	Brief-BESTest
Number of studies	26	48	16
Number of simples	32	54	18
Sample size total	1514	3876	1369
Sample size interrater	421	768	382
Sample size intra-rater	624	964	429

Since these coefficients are based on different assumptions, a reliability generalization meta-analysis has been separately performed for each coefficient in each of the three versions of the BESTest.

#### Evaluating the methodological quality of studies.

The quality of each study on a measurement property was independently assessed by two reviewers (ABMH and JALP) with the updated COSMIN (Consensus-based Standards for the selection of health Measurement Instruments) Risk of Bias checklist [58] regarding the 3 domains of measurement properties: reliability, validity and responsiveness. Each study was rated as follows: very good, adequate, doubtful or inadequate according to each specific item description. Methodological quality was rated with the lowest category obtained in the study. Specifically, internal consistency and inter- and intra-rater reliability have been assessed in each study included in this study because the main objective is to perform a meta-analysis of reliability generalisation.

In addition, the result of each study on a measurement property was rated against the updated criteria for good measurement properties using three values: sufficient (+), insufficient (−), or indeterminate (?). The details of how to score the quality of each study on a psychometric property and the result of each study on a psychometric property are fully described in the COSMIN guideline [58].

### Reliability estimates

Prior to meta-analysis, reliability coefficients were transformed to normalize their distributions and stabilize their variances. The alpha coefficient was transformed with the formula proposed by Bonnet [59], $L_{i}=\text{log}\left(1-\left|\hat{\alpha }\right|\right)$ , with log being the natural logarithm. The intraclass correlation coefficients to evaluate inter and intra-rater agreement were transformed with Fisher’s Z: $Z_{i}=0.5*log\left[\left(1+\hat{r}\right)/\left(1-\hat{r}\right)\right]$.

### Statistical analysis

Meta-analyses were conducted for internal consistency, inter or intra-rater reliability in the BESTest and in its two abbreviated versions. In all cases, a random-effects model was used and the confidential limits of 95% were calculated around the reliability coefficient with the improved method proposed by Hartung [60,61]. Between-study variance was estimated by restricted maximum likelihood [62].

To investigate heterogeneity of reliability coefficients in each meta-analysis, the Q statistic and the I^2^ index were calculated and a forest plot was created. If studies exhibited heterogeneity, a moderator analysis was then performed to identify study characteristics explaining why. Weighted ANOVA and simple meta-regression assuming a mixed-effects model were conducted for qualitative and quantitative moderators, respectively. A mixed-effects model was assumed using the improved method proposed by Knapp and Hartung to test the significance of moderating variables [63]. The proportion of variance explained for each moderating variable was estimated using the R2 index [64,65]. Statistical analysis was conducted with the metafor package in R [66].

To facilitate interpretation of results of each meta-analysis, the average reliability coefficients obtained with the Bonnet and Fisher Z transformations were back-transformed to the original metric of alpha coefficient and intraclass correlation, respectively. To determine whether publication bias could be a threat to validity of analytical results, funnel plots with the trim-and-fill imputation method of Duval and Tweedie [67] as well as the Egger test were applied [68,69].

## Results

### Study selection

**Fig 1:**
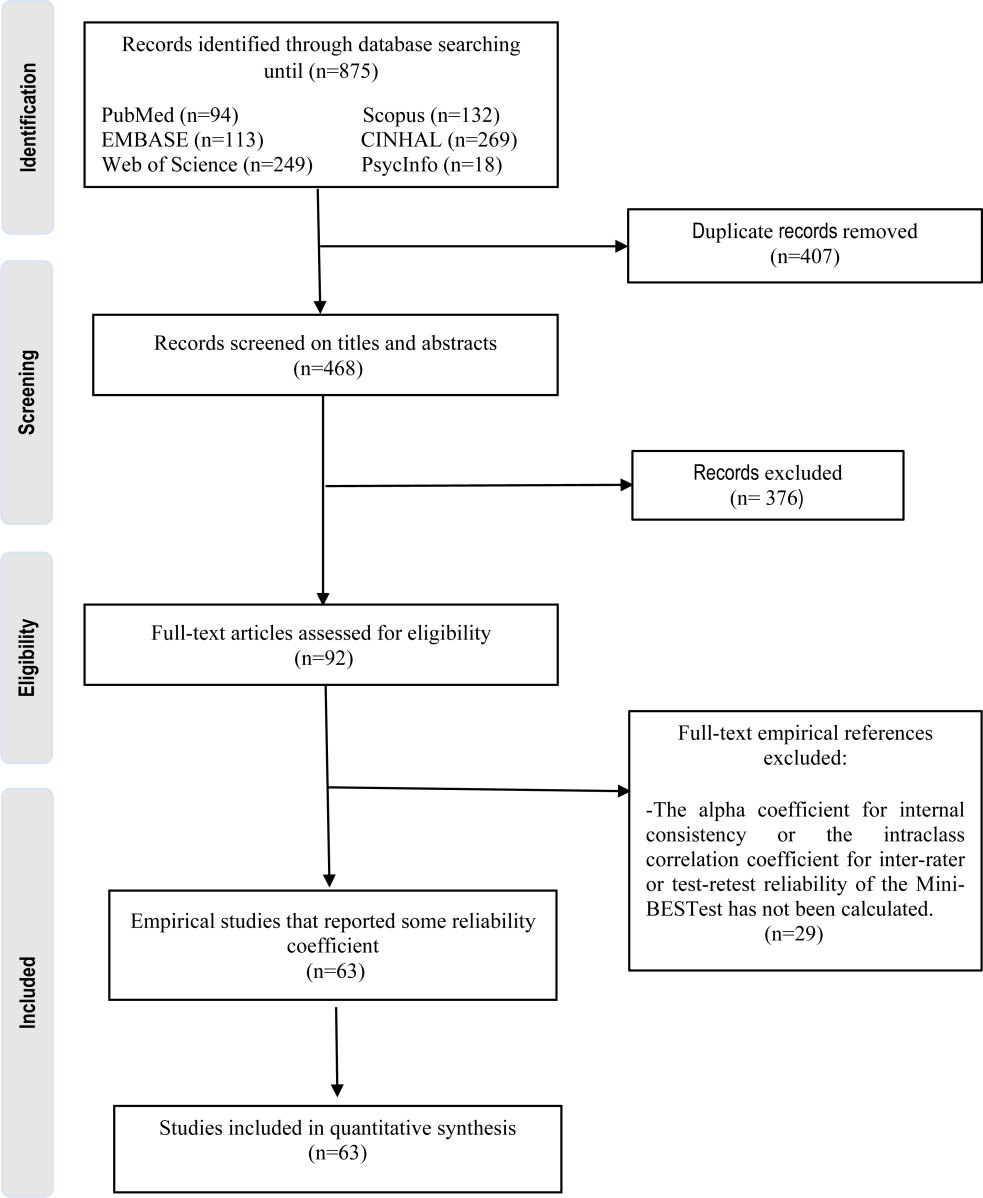
PRISMA flow diagram.

Fig 1 presents the PRISMA flow diagram describing the study selection process which met selection criteria. Overall, the search strategies identified a total of 875 articles. Following the removal of duplicates, 468 records screening of title/abstracts and 376 were excluded because they were narrative reviews, systematic reviews, scoping review, letters to editor, conference abstracts, study protocols, guidelines, studies not written in English and thesis or dissertations. In total, 92 full-text articles assessed for eligibility, of which 29 were excluded and a total of 63 [1,13,15,17–45,47–50,70–95] studies were eligible for the quantative analysis and were included in the current systematic review (Supplementary S2 Table). Supplementary S3 Table contains the completed PRISMA checklist.

### Descriptive characteristics of selected studies

Supplementary S4 Table presents the characteristics of 73 samples in 63 included studies. Some had coefficients such as internal consistency, inter and intra-rater (or test-retest) reliability [20,33,41,42,45,49,75,82,89,91], internal consistency and intra-rater (or test-retest) reliability [44,71], internal consistency and inter-rater reliability [32,43,76–78,87] or intra and inter-rater reliability [13,15,22,24,25,27,31,35,37,39,40,69,72–74,80,81,85,92,95] whereas others reported a single reliability coefficient such as internal consistency [17,18,36,46–48,83,88,90], intra-rater reliability (or test-retest) [19,21,23,26,28,30,34,38,70,84,86,93,94] or inter-rater reliability [1,29,79].

The sample size ranged from 10 to 709 [18,32]. Samples contained participants with or without illnesses. Some studies contained samples of participants with no pathology [13,19,25,26,40,43,45,72,86], while others included samples with pathology [1,15,17,18,20–24,26–31,33–35,37–39,41,42,44,47–49,69–71,73–78,80–87,89–95]. Most studies had samples from persons with a single illness such as stroke [22,24,29,33,37,38,77,82,85,88,91], multiple sclerosis [70,71,76,79,84] Parkinson’s disease [15,17,18,26,47,73–75,81,87], intellectual disability [30], chronic pain [23,83], spinal deformity [32], spinocerebellar ataxia [34], type 2 diabetes with peripheral neuropathy [20,21], spinal cord injury [80,90,93], total knee arthroplasty [41], cervical spondylotic myelopathy [42], cancer [69], chronic obstructive pulmonary disease [27,92], end-stage renal disease [28]. The most common pathology was stroke. Few studies included participants with different pathologies in their samples [1,31,35,39,44,46,48,49,78,89,95].

#### The methodological quality of studies.

Four, twenty-four and six studies (seven samples) evaluated the internal consistency of the BESTest [32,41,43,71], Mini-BESTest [17,20,33,36,38,41,43–48,75–78,82,83,87–91,95] and Brief-BESTest [41,42,48,49,78,91], respectively. All studies were of very good and sufficient quality. Only one had inadequate and sufficient quality for Mini-BESTest [17] (Supplementary S5 Table).

Regarding the BESTest, eighteen and twenty-two studies (nineteen and twenty-four samples) assessed inter-rater[1,13,22,24,25,27,30,32,35,40–43,69,72–74,78] and intra-rater/test-retest reliability [13,19,22–28,34,35,40–42,69,70–74,86,94], respectively. All studies were of doubtful and sufficient quality. Only one was of very good and sufficient quality [35] (Supplementary S5 Table).

Thirty and thirty-one studies assessed the inter-rater [13,15,20,25,27,29,31,33,35,39–43,45,69,71,73,75–82,85,87,89,95] and intra-rater/test-retest reliability [13,15,19–21,25–28,31,33–35,38–40,42,44,45,69,73,75,80–82,84–86,89,93,95] of the Mini-BESTest, respectively. Most studies were of doubtful and sufficient quality. Three studies had adequate inter-rater and intra-rater/test-retest reliability [29,33,80] and 4 studies very good for both types of reliability [35,76,81,95]. Only one study had inadequate inter-rater and intra-rater/test-retest reliability [89].

Finally, regarding the Brief-BESTest, thirteen and fourteen studies assessed inter-rater [13,15,25,27,37,41,42,49,69,78,85,91,92] and intra-rater/test-retest [13,15,25,27,28,34,37,41,42,49,69,85,91,92] quality, respectively. All studies were of doubtful and sufficient quality.

### Mean reliability and heterogeneity

Reliability studies using the BESTest scale and its abbreviated versions Mini-BESTest and Brief-BESTest must collect one or more reliability coefficients (inter rater agreement or intra-rater) or an internal consistency coefficient (alpha). Separate meta-analyses were performed for each reliability coefficient and internal consistency in each of the three scales. Alpha coefficients were reported in only four studies for BESTest [32,41,43,71]. This low number of reported coefficients did not allow meta-analysis to be carried out. Thus, a total of 8 meta-analyses were conducted.

**Fig 2:**
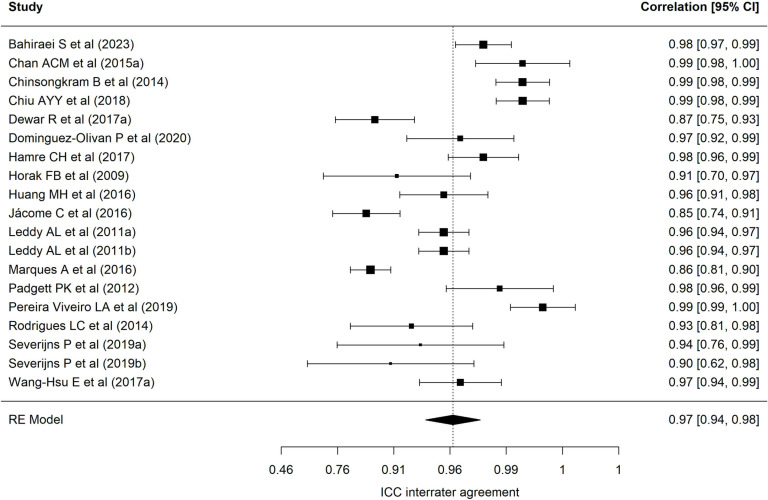
Forest plot BESTest ICC inter-rater agreement.

**Fig 3:**
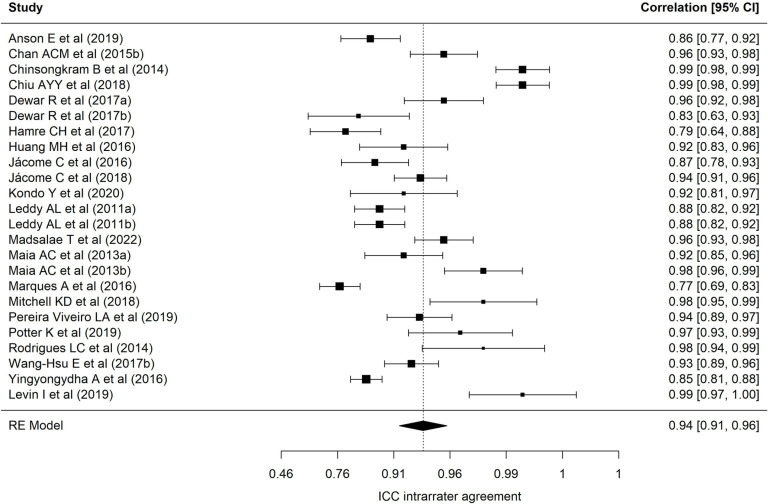
Forest plot BESTest ICC intra-rater agreement.

**Fig 4:**
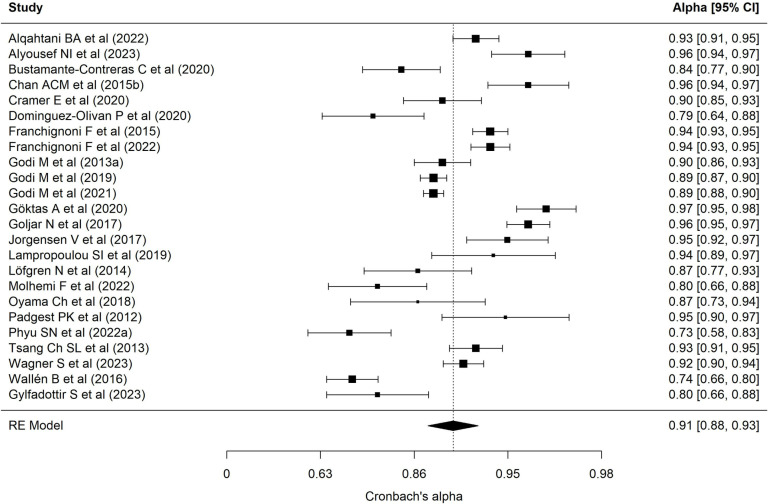
Forest plot Mini-BESTest Cronbach´s alpha.

**Fig 5:**
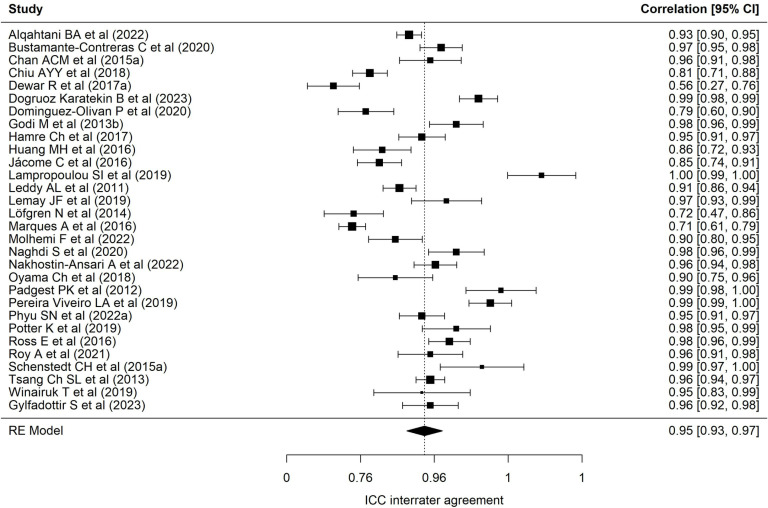
Forest plot Mini-BESTest inter-rater agreement.

**Fig 6:**
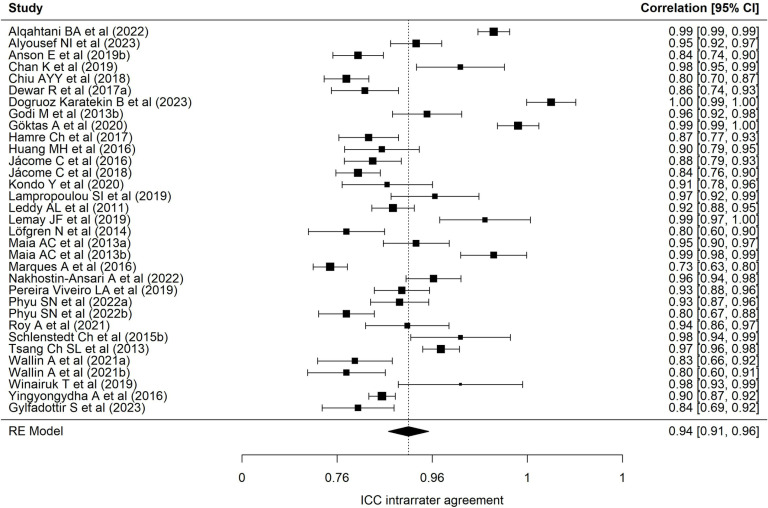
Forest plot Mini-BESTest intra-rater agreement.

**Fig 7:**
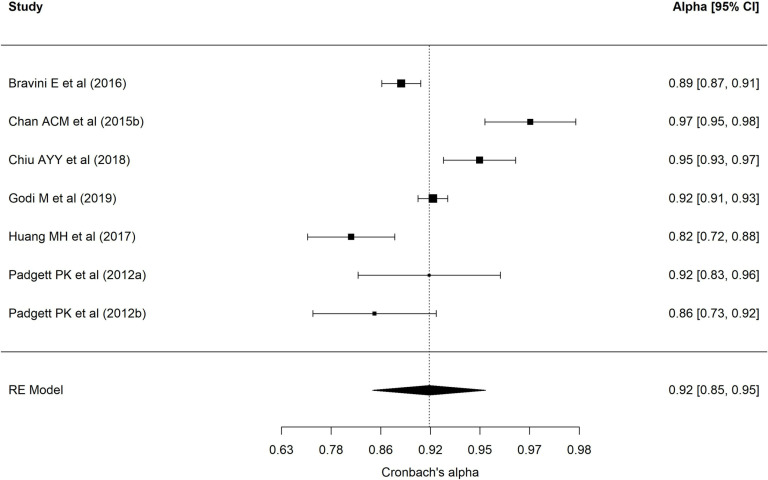
Forest plot Brief-BESTest Cronbach´s alpha.

**Fig 8:**
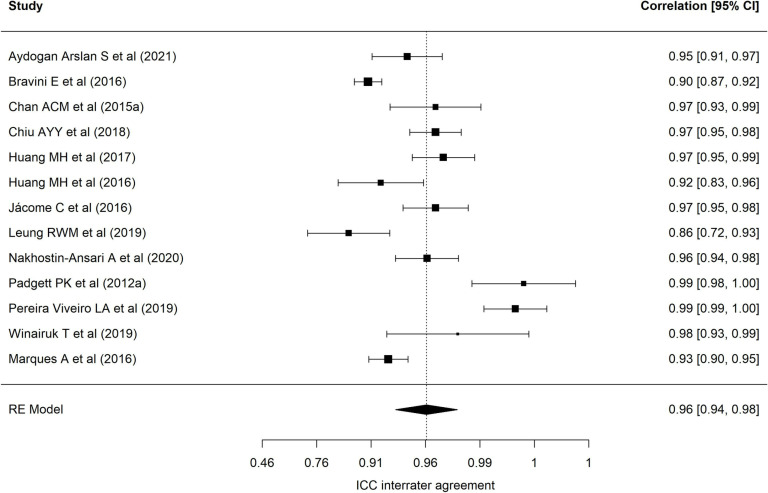
Forest plot Brief-BESTest inter-rater agreement.

**Fig 9:**
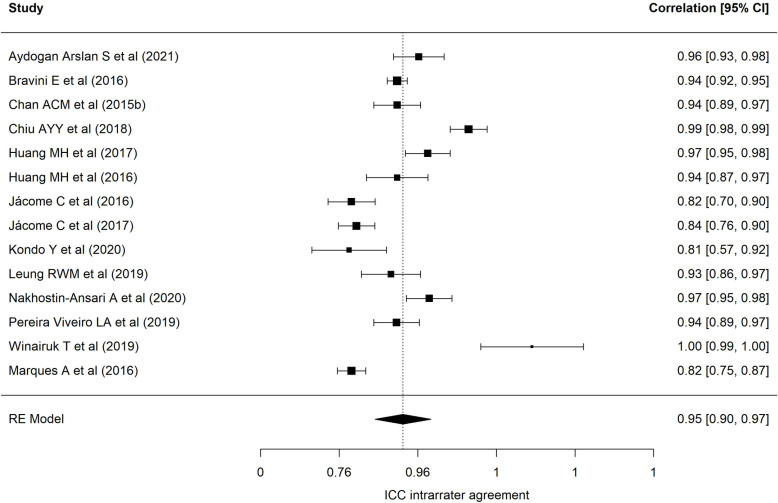
Forest plot Brief-BESTest intra-rater agreement.

Table 2 presents results of each of the eight meta-analyses performed. Regarding the BESTest scale, nineteen samples reported a mean interrater ICC of 0.97 (95% CI = 0.94–0.98), with wide heterogeneity (90.69%) [1,13,22,24,25,27,30,32,35,40–43,69,72–74,78]. Fig 2 presents a forest plot of these coefficients. The 24 samples that reported an intra-rater ICC (Fig 3, forest plot) showed a mean ICC of 0.94 (95%CI: 0.91–0.96) with heterogeneity of 89.70% [13,19,22–28,34,35,40–42,69,70–74,86,94]. Twenty-four samples reported an alpha coefficient of internal consistency for the Mini-BESTest [17,20,33,36,38,41,43–48,75–78,82,83,87–91,95] (Fig 4; forest plot). This meta-analysis reported a mean alpha coefficient of 0.91 (95%CI: 0.89-0.94) with heterogeneity of 94.42%. As for inter-rater agreement, 30 reported a CCI; the mean ICC in meta-analysis was 0.95 (95%CI: 0.92–0.97) with heterogeneity of 94.67% [13,15,20,25,27,29,31,33,35,39–43,45,69,71,73,75–82,85,87,89,95] (Fig 5; forest plot). For intra-rater agreement, reported by 33 samples, in the Mini-BESTest, the mean ICC was 0.94 (95%CI: 0.91-0.96) with heterogeneity of 3.93% [13,15,19–21,25–28,31,33–35,38–40,42,44,45,69,73,75,80–82,84–86,89,93,95] (Fig 6; forest plot). Finally, on the Brief-BESTest scale, 7 samples reported an alpha coefficient, whose mean was 0.92 (95%CI: 0.85-0.95) with heterogeneity of 92.93% [41,42,48,49,78,91] (Fig 7; forest plot); mean ICC, in 13 samples, for inter-rater agreement was 0.97 (95%CI: 0.94–0.98) and heterogeneity 90.21% [13,15,25,27,37,41,42,49,69,78,85,91,92] (Fig 8; forest plot), while the mean ICC, in 14 samples, for intra-rater agreement was 0.95 (95%CI: 0.90–0.98) with heterogeneity of 93.97% [13,15,25,27,28,34,37,41,42,49,69,85,91,92] (Fig 9; forest plot).

**Table 2:** Synthesis of the reliability estimates obtained from the BESTest, Mini-BESTest and brief-BESTest.

Scale	K	Min	Max	Mean	95%CI	Q	P	I^2^
BESTest								
Alpha	4	0.70	0.98	0.92	0.34–0.99	41.673	<0.001	93.12
ICCinterrater	19	0.85	0.99	0.97	0.94–0.98	212.999	<0.001	89.93
ICCintrarater	24	0.77	0.99	0.94	0.91–0.96	311.676	<0.001	92.00
Mini-BESTest								
Alpha	24	0.73	0.97	0.91	0.88–0.93	306.620	<0.001	94.81
ICCinterrater	30	0.56	1.00	0.95	0.93–0.97	431.820	<0.001	93.88
ICCintrarater	33	0.73	1.00	0.94	0.91–0.96	654.844	<0.001	94.20
Brief-BESTest								
Alpha	7	0.86	0.97	0.92	0.85–0.95	49.806	<0.001	92.93
ICCinterrater	13	0.86	0.99	0.96	0.94–0.98	126.779	<0.001	90.89
ICCintrarater	14	0.81	1.00	0.95	0.90–0.97	180.843	<0.001	94.71

K: number of studies; Min: minimum reliability coefficient; Max: maximum reliability coefficient; Q: Cochran’s statistic to test the null hypothesis of homogeneity; p: probability value; I^2^ = heterogeneity index.

### Moderator analyses

The eight meta-analyses found sufficient heterogeneity of ICC and alpha coefficients which led to a moderator analysis to partly explain heterogeneity of reliability estimates.

#### BESTest scale.

Table 3 presents the results of simple regression meta-analyses for continuous moderators on intraclass correlation (inter-rater agreement) on the BESTest scale. In this case, only the mean scores exhibited statistical significance with intraclass correlation (p = 0.014, R^2^ =  45.83). The negative sign of the regression slope for mean scores indicated a decrease in intraclass correlation as the sample mean increased.

**Table 3:** Results of the simple meta-regression applied on intraclass correlation (interrater agreement) taking continuous moderator variables as predictors in the BESTest.

Predictor variable	K	b_j_	F	P	Q_E_	R^2^
Mean scores	12	−0.025	8.777	0.014	75.473***	45.83
SD of scores	12	0.018	0.983	0.345	174.965***	0
Sample size	19	0	0.001	0.973	189.137***	0
Mean age (years)	19	0.003	0.286	0.6	211.519***	0
SD of age (years)	19	0.033	0.673	0.423	197.776***	0
Gender (% females)	18	−0.002	0.06	0.81	182.463***	0
Year of study	19	0.019	0.283	0.602	206.874***	0
Mean of disorder history (years)	4	−0.082	17.062	0.054	1.009	100
Experience with BESTest (years)	10	−0.022	0.63	0.45	89.840***	0
Number of raters	17	−0.019	0.056	0.816	196.847***	0
Sample size ICC (inter-rater)	17	0.011	0.926	0.351	202.629***	0

k: number of studies; b_j_: regression coefficient of each predictor; F: Knapp-Hartung’s statistics for testing the significance of the predictor (degree freedom 1 and k-2); p: probability level for the F statistic; Q_E_: statistics for testing the model misspecification; R^2^: proportion of variance accounted for by the predictor. Regression coefficients were back-transformed to the original metric.

ANOVA weighted with respect to qualitative variables are shown in Table 4. Significant differences between intraclass correlations were found depending on the continent the study proceeded from (p = 0.016, R^2^ = 51.06) with 51.06% of explained variance. Thus, the lowest inter-rater ICC (ICC =  0.87, n = 1) was obtained in Australia and the highest (on average) in Asia (ICC =  0.988, n = 4).

**Table 4:** Results of the ANOVAs for the qualitative methodological and substantive moderator variables on intraclass correlation (inter-rater agreement) in the BESTest.

Variable	K	ICC_+_	95% CI		ANOVA results
			LL	LU	
Test version					F(1,17) = 0.004; p = 0.952
Original	9	0.966	0.932	0.984	R^2^ = 0.0
Adaptation	10	0.965	0.931	0.983	Q_w_ = 210.598; p < 0.001
Type of design					F(1,17) = 0.018; p = 0.894
Observational	18	0.966	0.943	0.979	R^2^ = 0.0
Experimental	1	0.970	0.765	0.996	Q_w_ = 212.872;p < 0.001
Study focus					F(1,17) = 0.469;p = 0.503
Psychometric	14	0.969	0.945	0.983	R^2^ = 0.0
Applied	5	0.956	0.887	0.983	Q_w_ = 209.000;p < 0.001
Continent					F(4,14) = 4.456;p = 0.016
Asia	4	0.988	0.974	0.995	R^2^ = 51.06
Australia	1	0.870	0.483	0.973	Q_e_ = 63.856;p < 0.001
Europe	6	0.933	0.869	0.966	
North America	6	0.964	0.930	0.981	
South America	2	0.982	0.941	0.994	
Population target 1					F(3,15) = 0.731;p = 0.549
Children and adolescents	1	0.870	0.242	0.984	R^2^ = 0.0
Adults(19–65)	1	0.980	0.847	0.998	Q_w_ = 193,394;p < 0.001
Adults > 65 years	1	0.970	0.762	0.997	
Mixed (Adults 19–99)	16	0.967	0.944	0.981	
Population target 2					F(3,15) = 1.803;p = 0.190
Clinical	12	0.969	0.945	0.983	R^2^ = 16.62
Non-institucionalized	4	0.933	0.828	0.975	Q_w_ = 117.470,p < 0.001
Institucionalized	1	0.993	0.952	0.999	
Clinical + non-institucionalized	2	0.966	0.848	0.993	
Rater formation					F(2,15) = 0.034;p = 0.967
Physiotherapist	13	0.963	0.930	0.981	R^2^ = 0.0
Physiotherapist (ended + no ended)	4	0.967	0.900	0.990	Q_w_ = 201.688;p < 0.001
Physiotherapist + MD	1	0.970	0.735	0.997	
Disease					F(8,10) = 0.882;p = 0.563
No disease	5	0.956	0.888	0.985	R^2^ = 0.0
Parkinson´s disease	1	0.960	0.824	0.991	Q_w_ = 110.635;p < 0.001
Stroke	3	0.972	0.896	0.993	
Cervical spondylotic myelopathy	1	0.990	0.913	0.999	
Chronic obstructive pulmonary disease	1	0.850	0.134	0.983	
Total knee artroplasty	1	0.990	0.901	0.998	
Diverse neurological diagnoses	3	0.972	0.892	0.993	
Spinal deformity	1	0.922	0.572	0.988	

K: number of studies; ICC_+_ : Mean intraclass correlation; LL and LU: lower and upper 95% confidence limits for ICC; F: Knapp-Hartung’s statistic for testing the significance of the moderator variable; Q_W_: statistic for testing the model misspecification; R^2^: proportion of variance accounted for by the moderator. The average reliability coefficients and their confidence limits were back-transformed to the original metric.

Meta-analyses for continuous moderators of intra-rater ICCs are shown in Table 5. Only the mean of disorder history (years) obtained marginal statistical significance in the intra-class intra-rater correlation (p = 0.076, R^2^ = 79.28). Mean of disorder history could also be considered marginally significant, but the number of studies is very small.

**Table 5:** Results of the simple meta-regression applied on intraclass correlation (intra-rater agreement) taking continuous moderator variables as predictors in the BESTest.

Predictor variable	K	b_j_	F	P	Q_E_	R^2^
Mean scores	18	−0.016	2.367	0.144	235.152***	8.88
SD of scores	18	0.029	2.999	0.103	240.258***	11.1
Sample size	24	−0.004	3.275	0.084	245.540***	9.84
Mean age (years)	24	−0.007	1.712	0.204	272.229***	4.03
SD of age (years)	24	0.016	0.252	0.621	303.693***	0
Gender (% females)	23	−0.009	2.307	0.144	247.134***	7.01
Year of study	24	0.025	0.412	0.527	299.719***	0
Mean of disorder history (years)	6	0.052	5.685	0.076	5.834	79.28
SD of disorder history (years)	5	0.102	3.161	0.174	4.552	82.48
Experience with BESTest (years)	12	−0.03	1.154	0.308	132.801***	3.01
Interval intra-rater	21	−0.018	1.666	0.212	260.302***	3.12
Sample size ICC (intra-rater)	22	−0.008	1.39	0.252	288.739***	1.15

k: number of studies; b_j_: regression coefficient of each predictor; F: Knapp-Hartung’s statistics for testing the significance of the predictor (degree freedom 1 and k-2); p: probability level for the F statistic; Q_E_: statistics for testing the model misspecification; R^2^: proportion of variance accounted by the predictor. Regression coefficients were back-transformed to the original metric.

Weighted ANOVA for the categorical moderating variables on the ICCs (intra-rater) of the BESTest scale are shown in Table 6. The continent the study proceeded from obtained marginal statistical significance in the intra-class intra-rater correlation (p = 0.064, R^2^ = 27.45) with 27.45% of explained variance. Thus, the lowest intra-rater ICC (ICC =  0.872, n = 5) was obtained in Europe and the highest (on average) in Asia (ICC =  0.969, n = 5).

**Table 6:** Results of the ANOVAs for the qualitative methodological and substantive moderator variables on intraclass correlation (intra-rater agreement) in the BESTest.

Variable	K	ICC_+_	95% CI		ANOVA results
			LL	LU	
Test version					F(1,23) = 0.109;p = 0.927
Original	12	0.942	0.894	0.968	R^2^ = 0.0
Adaptation	12	0.944	0.899	0.969	Q_W_ = 309.531;p < 0.001
Type of design					F(1,22) = 0.045;p = 0.835
Observational	23	0.943	0.913	0.963	R^2^ = 0.0
Experimental	1	0.930	0.551	0.991	Q_W_ = 311.649;p < 0.001
Study focus					F(1,22) = 1.428;p = 0.245
Psychometric	17	0.951	0.920	0.970	R^2^ = 1.4
Applied	7	0.918	0.831	0.961	Q_W_ = 288.031;p < 0.001
Continent					F(5,18) = 2.571;p = 0.064
Asia	5	0.969	0.934	0.986	R^2^ = 27.45
Australia	2	0.920	0.724	0.978	Q_W_ = 224.975;p < 0.001
Europe	5	0.872	0.734	0.941	
North America	8	0.927	0.860	0.963	
South America	4	0.961	0.904	0.985	
Population target 1					F(3,20) = 1.392;p = 0.274
Children and adolescents	2	0.919	0.685	0.981	R^2^ = 3.63
Adults (19–65)	2	0.986	0.936	0.997	Q_W_ = 287.167;p < 0.001
Adults > 65 years	1	0.930	0.580	0.990	
Mixed (Adults 19–99)	19	0.938	0.902	0.961	
Population target 2					F(3,20) = 0.834,p = 0.491
Clinical	15	0.954	0.921	0.973	R^2^ = 0.00
No-institucionalized	7	0.908	0.807	0.957	Q_W_ = 241.522;p < 0.001
Institucionalized	1	0.939	0.593	0.992	
Clinical + no-institucionalized	1	0.960	0.720	0.995	
Rater formation					F(2,16) = 0.031;p = 0.970
Physiotherapist	16	0.947	0.903	0.971	R2 = 0.0
Physiotherapist (ended + no ended)	3	0.947	0.812	0.986	Q_W_ = 252.162;p < 0.001
Physiotherapist + MD	1	0.930	0.467	0.993	
Disease					F(11,12) = 2.145;p = 0.103
No disease	8	0.912	0.843	0.951	R^2^ = 39.51
Parkinson´s disease	2	0.894	0.738	0.959	Q_W_ = 89.128;p < 0.001
Stroke	3	0.976	0.931	0.991	
Multiple sclerosis	2	0.976	0.908	0.994	
Cervical spondylotic myelopathy	1	0.990	0.947	0.998	
Spinocerebellar ataxia	1	0.920	0.556	0.988	
Chronic obstructive pulmonary disease	1	0.870	0.433	0.976	
End-stage renal disease	1	0.940	0.713	0.989	
Chronic pain	1	0.960	0.798	0.993	
Total knee artroplasty	1	0.960	0.792	0.993	
Diverse neurological diagnoses	1	0.790	0.193	0.960	
sCerebral palsy	1	0.990	0.934	0.999	

K: number of studies; ICC_+ _: Mean intraclass correlation; LL and LU: lower and upper 95% confidence limits for ICC; F: Knapp-Hartung’s statistic for testing the significance of the moderator variable; Q_W_: statistic for testing the model misspecification; R^2^: proportion of variance accounted for by the moderator. The average reliability coefficients and their confidence limits were back-transformed to the original metric.

#### Mini-BESTest scale.

The results of applying simple meta-regressions to the continuous moderating variables to the alpha coefficient in the Mini-BESTest are shown in Table 7. Standard deviation of scores was marginally significant (p = 0.073; R^2^ = 15.29) with a positive regression weight indicating that an increase in standard deviation of the sample means an increase in the alpha coefficient. The gender moderator was significant (p = 0.042; R^2^ = 14.94%) a negative weight signifying an increase in the alpha coefficient when the number of women decreased.

**Table 7:** Results of the simple meta-regression applied on alpha coefficient taking continuous moderator variables as predictors in the Mini-BESTest.

Predictor variable	K	b_j_	F	P	Q_E_	R^2^
Mean scores	17	−0.008	0.3928	0.5402	196.256***	0.00
SD of scores	15	0.104	3.796	0.0733	188.032***	15.29
Sample size	24	0.000	0.012	0.9141	282.492***	0.00
Mean age (years)	24	−0.006	0.218	0.6452	292.280***	0.00
SD of age (years)	24	0.030	1.141	0.2969	279.309***	2.15
Gender (% females)	24	−0.016	4.690	0.0415	280.998***	14.94
Year of study	24	−0.037	0.957	0.3387	300.558***	0.00
Mean of disorder history (years)	8	−0.084	2.379	0.1740	60.517***	18.74
SD of disorder history (years)	8	−0.157	3.755	0.1008	49.810***	33.41
Experience with Mini-BESTest (years)	5	−0.043	0.176	0.7033	50.081***	0.00

k: number of studies; b_j_: regression coefficient of each predictor; F: Knapp-Hartung’s statistics for testing the significance of the predictor (degree freedom 1 and k-2); p: probability level for the F statistic; Q_E_: statistics for testing the model misspecification; R^2^: proportion of variance accounted by the predictor. Regression coefficients were back-transformed to the original metric.

Weighted ANOVA for categorical variables on internal consistency (alpha coefficient) on the Mini-BESTest scale are shown in Table 8. The moderator of disease was marginally significant (p = 0.088; R^2^ = 33.24) with total knee arthroplasty than other diseases. The lowest coefficient was obtained in patients with type 2 diabetes.

**Table 8:** Results of the ANOVAs for the qualitative methodological and substantive moderator variables on alpha coefficient in the Mini-BESTest.

Variable	K	Α	95% CI		ANOVA results
			LL	LU	
Test version					F(2,21) = 1.138;p = 0.340
Original	4	0.923	0.853	0.960	R^2^ = 0.70
Adaptation	19	0.904	0.867	0.929	Q_W_ = 258.918;p < 0.001
Multicentric	1	0.960	0.859	0.989	
Type of design					F(1,22) = 0.157;p = 0.696
Observational	23	0.910	0.881	0.932	R^2^ = 0.0
Experimental	1	0.930	0.743	0.981	Q_W_ = 304.065;p < 0.001
Study focus					F(1,21) = 0.402;p = 0.533
Psychometric	22	0.908	0.878	0.931	R^2^ = 0.00
Applied	2	0.932	0.829	0.973	Q_W_ = 302.455;p < 0.001
Continent					F(3,20) = 0.595;p = 0.626
Asia	8	0.921	0.873	0.952	R^2^ = 0.0
Europe	14	0.905	0.863	0.934	Q_W_ = 277.866;p < 0.001
North America	1	0.949	0.777	0.988	
South America	1	0.845	0.396	0.960	
Population target 1					F(1,22) = 0.004;p = 0.951
Adults (19–65)	2	0.913	0.772	0.967	R^2^ = 0.0
Mixed (Adults 19–99)	22	0.911	0.881	0.933	Q_W_ = 304.838;p < 0.001
Population target 2					F(2,21) = 0.484;p = 0.623
Clinical	21	0.911	0.881	0.934	R^2^ = 0.0
No-institucionalized	2	0.884	0.697	0.955	Q_W_ = 303.987;p < 0.001
Clinical + no-institucionalized	1	0.949	0.779	0.988	
Rater formation					F(2,12) = 0.557;p = 0.587
Physiotherapist	13	0.895	0.844	0.929	R^2^ = 0.0
Physiotherapist (ended + no ended)	1	0.800	0.1334	0.954	Q_W_ = 149.336;p < 0.001
Other	1	0.845	0.363	0.962	
Disease					F(8,15) = 2.214;p = 0.088
No disease	2	0.886	0.746	0.949	R^2^ = 33.24
Parkinson´s disease	5	0.873	0.792	0.923	Q_W_ = 156.898;p < 0.001
Stroke	6	0.939	0.902	0.962	
Multiple sclerosis	1	0.800	0.344	0.939	
Spinal cord injury	1	0.950	0.843	0.984	
Type 2 diabetes	1	0.730	0.149	0.914	
Chronic pain	1	0.920	0.767	0.973	
Total knee artroplasty	2	0.960	0.911	0.982	
Diverse neurological diagnoses	5	0.907	0.847	0.944	

K: number of studies; ICC_+ _: Mean coefficient alpha; LL and LU: lower and upper 95% confidence limits for α; F: Knapp-Hartung’s statistic for testing the significance of the moderator variable; Q_W_: statistic for testing the model misspecification; R^2^: proportion of variance accounted for by the moderator. The average reliability coefficients and their confidence limits were back-transformed to the original metric.

Regarding ICCs (inter-rater agreement), simple meta-regressions for continuous moderators are shown in Table 9. In this case, the raters’ experience variable was significant (p = 0.019; R^2^ = 32.04) with a negative regression weight, indicating that an increase in the experience of evaluators led to a decrease in inter-rater ICC. The rest of variables obtained no significant results.

**Table 9:** Results of the simple meta-regression applied on intraclass coefficient (interrater agreement) taking continuous moderator variables as predictors in the Mini-BESTest.

Predictor variable	K	b_j_	F	P	Q_E_	R^2^
Mean scores	23	0.0017	0.0381	0.8472	334.470***	0.00
SD of scores	22	0.0753	23.889	0.1379	327.172***	5.95
Sample size	30	−0.0053	20.885	0.1595	384.843***	3.88
Mean age (years)	30	0.0074	0.6797	0.4167	431.777***	0.00
SD of age (years)	30	0.0342	20.285	0.1654	404.133***	4.34
Gender (% females)	27	−0.0029	0.1517	0.7002	411.734***	0.00
Year of study	30	0.0074	0.0386	0.8457	422.677***	0.00
Mean of disorder history (years)	13	0.0303	0.5135	0.4886	99.965***	0.00
SD of disorder history (years)	13	0.0753	17.850	0.2085	93.769***	6.64
Experience with Mini-BESTest (years)	15	−0.0590	70.883	0.0195	123.183***	32.04
Number of raters	28	−0.0046	0.0035	0.9536	423.958***	0.00
Sample size ICC (inter-rater)	26	0.0056	0.2810	0.6009	373.931***	0.00

k: number of studies; b_j_: regression coefficient of each predictor; F: Knapp-Hartung’s statistics for testing the significance of the predictor (degree freedom 1 and k-2); p: probability level for the F statistic; Q_E_: statistics for testing the model misspecification; R^2^: proportion of variance accounted by the predictor. Regression coefficients were back-transformed to the original metric.

The weighted ANOVAs for the categorical variables on the Mini-BESTest scale for the ICC (inter-rater agreement) are shown in Table 10. The moderator of population type was significant (p = 0.013; R^2^ = 28.65) with the normal institutionalized population indicating a higher mean reliability (ICC_+_ = 0.992) than the mixed population (ICC_+_ = 0.982) or clinical population (ICC_+_ = 0.959). The lowest coefficient was obtained in the normal, non-institutionalized population (ICC_+_ = 0.79).

**Table 10:** Results of the ANOVAs for the qualitative methodological and substantive moderator variables on intraclass coefficient (interrater agreement) in the Mini-BESTest.

Variable	K	ICC_+_	95% CI		ANOVA results
			LL	LU	
Test version					F(1,28) = 0.052;p = 0.822
Original	11	0.950	0.891	0.978	R^2^ = 0.0
Adaptation	19	0.955	0.912	0.976	Q_W_ = 430.795;p < 0.001
Type of design					F(1,28) = 0.036;p = 0.852
Observational	27	0.953	0.922	0.972	R^2^ = 0.0
Experimental	3	0.959	0.819	0.991	Q_W_ = 424.038;p < 0.001
Study focus					F(1,28) = 0.475;p = 0.496
Psychometric	26	0.951	0.918	0.970	R^2^ = 0.0
Applied	4	0.969	0.886	0.992	Q_W_ = 431.326;p < 0.001
Continent					F(4,25) = 1.492;p = 0.235
Asia	11	0.951	0.896	0.977	R^2^ = 7.27
Australia	1	0.560	−0.569	0.957	Q_W_ = 333.590;p < 0.001
Europe	10	0.950	0.890	0.977	
North America	6	0.965	0.901	0.988	
South America	2	0.985	0.911	0.997	
Population target 1					F(2,27) = 2.067;p = 0.146
Children and adolescents	1	0.560	−0.564	0.957	R^2^ = 7.38
Adults (19–65)	2	0.951	0.741	0.992	Q_W_ = 390.071;p < 0.001
Mixed (Adults 19–99)	27	0.958	0.932	0.974	
Population target 2					F(3,26) = 4.344;p = 0.013
Clinical	23	0.959	0.934	0.974	R^2^ = 28.65
Non-institucionalized	4	0.790	0.473	0.926	Q_W_ = 273.216;p < 0.001
Institucionalized	1	0.992	0.928	0.999	
Clinical + non-institucionalized	2	0.982	0.909	0.996	
Rater formation					F(2,25) = 0.719;p = 0.497
Physiotherapist	24	0.943	0.004	0.966	R^2^ = 0.0
Physiotherapist (ended + no ended)	1	0.960	0.548	0.997	Q_W_ = 350.308;p < 0.001
Other (sport scientist)	3	0.977	0.898	0.995	
Disease					F(9,20) = 0.863;p = 0.572
No disease	5	0.887	0.665	0.965	R^2^ = 0.0
Parkinson´s disease	5	0.949	0.837	0.985	Q_W_ = 278.587;p < 0.001
Stroke	6	0.967	0.901	0.990	
Multiple sclerosis	3				
Spinal cord injury	1	0.960	0.505	0.998	
Cervical spondylotic myelopathy	1	0.810	−0.210	0.986	
Type 2 diabetes	1	0.950	0.442	0.997	
Chronic obstructive pulmonary disease	1	0.850	−0.098	0.989	
Total knee artroplasty	1	0.960	0.505	0.998	
Diverse neurological diagnoses	6	0.980	0.938	0.993	

K: number of studies; ICC_ + _: Mean intraclass correlation; LL and LU: lower and upper 95% confidence limits for ICC; F: Knapp-Hartung’s statistic for testing the significance of the moderator variable; Q_W_: statistic for testing the model misspecification; R^2^: proportion of variance accounted for by the moderator. The average reliability coefficients and their confidence limits were back-transformed to the original metric.

The simple regression meta-analyses of the continuous moderating variables of the Mini-BESTest for the ICC (intra-rater agreement) are shown in Table 11. In this case, the mean history of the disorder was significant (p = 0.024, R^2^ = 35.51) with a negative weight, indicating an increase in number of years with the disorder suffered by patients implied a decrease in intra-rater agreement.

**Table 11:** Results of the simple meta-regression applied on intraclass coefficient (intra-rater agreement) taking continuous moderator variables as predictors in the Mini-BESTest.

Predictor variable	K	b_j_	F	P	Q_E_	R^2^
Mean scores	28	−0,028	2,111	0,158	576.256***	4,16
SD of scores	27	0,034	0,709	0,408	577.346***	0
Sample size	33	0	0,001	0,991	654.800***	0
Mean age (years)	33	−0,001	0,004	0,949	641.539***	0
SD of age (years)	33	0,035	1,466	0,235	646.226***	1,46
Gender (% females)	30	−0,008	1,772	0,194	561.207***	3,05
Year of study	33	0,015	0,206	0,653	598.987***	0
Mean of disorder history (years)	13	−0,117	6,815	0,024	132.162***	35,51
SD of disorder history (years)	13	−0,106	2,205	0,166	195.091***	10,11
Experience with Mini-BESTest (years)	12	−0,025	1,730	0,218	76.025***	6,48
Interval intra-rater	28	0,004	0,056	0,815	595.809***	0
Number of raters	31	0,210	1,699	0,203	572.348***	2,9
Sample size ICC (intra-rater)	31	−0,002	0,030	0,863	633.717***	0

k: number of studies; b_j_: regression coefficient of each predictor; F: Knapp-Hartung’s statistics for testing the significance of the predictor (degree freedom 1 and k-2); p: probability level for the F statistic; Q_E_: statistics for testing the model misspecification; R^2^: proportion of variance accounted by the predictor. Regression coefficients were back-transformed to the original metric.

The weighted ANOVA for categorical variables in the Mini-BESTest for ICC (intra-rater agreement) is shown in Table 12. No moderating variables were significant in this case.

**Table 12:** Results of the ANOVAs for the qualitative methodological and substantive moderator variables on intraclass coefficient (intra-rater agreement) in the Mini-BESTest.

Variable	K	ICC_+_	95% CI		ANOVA results
			LL	LU	
Test version					F(1,31) = 0.573;p = 0.455
Original	10	0.926	0.846	0.965	R^2^ = 0.00
Adaptation	23	0.947	0.913	0.968	Q_W_ = 638.833;p < 0.001
Type of design					F(2,30) = 1.188;p = 0.319
Observational	29	0.940	0.907	0.961	R^2^ = 0.90
Cuasi-experimental	1	0.800	−0.103	0.980	Q_W_ = 618.181;p < 0.001
Cohort study	3	0.972	0.887	0.993	
Study focus					F(1,31) = 0.011;p = 0.916
Psychometric	28	0.942	0.909	0.963	R^2^ = 0.0
Applied	5	0.938	0.824	0.979	Q_W_ = 631.779;p < 0.001
Continent					F(4,28) = 1.866;p = 0.144
Asia	12	0.964	0.930	0.981	R^2^ = 10.99
Australia	1	0.860	0.124	0.986	Q_W_ = 479.562;p < 0.001
Europe	11	0.889	0.786	0.944	
North America	6	0.946	0.865	0.979	
South America	3	0.967	0.881	0.991	
Population target 1					F(2,30) = 0.467;p = 0.631
Children and adolescents	1	0.860	0.047	0.988	R^2^ = 0.0
Adults (19–65)	2	0.909	0.566	0.984	Q_W_ = 646.671;p < 0.001
Mixed (Adults 19–99)	30	0.945	0.915	0.965	
Population target 2					F(3,29) = 0.196;p = 0.898
Clinical	25	0.945	0.910	0.967	R^2^ = 0.0
No-institucionalized	6	0.935	0.830	0.976	Q_W_ = 639.795;p < 0.001
Institucionalized	1	0.933	0.399	0.994	
Clinical+no-institucionalized	1	0.870	0.068	0.989	
Rater formation					F(2,21) = 0.810;p = 0.458
Physiotherapist	21	0.935	0.888	0.962	R^2^ = 0.0
Physiotherapist (ended + no ended)	1	0.840	−0.065	0.987	Q_W_ = 454.862;p < 0.001
Other	2	0.973	0.842	0.996	
Disease					F(11,21) = 0.809;p = 0.632
No disease	7	0.935	0.839	0.975	R^2^ = 0.0
Parkinson´s disease	5	0.942	0.828	0.982	Q_W_ = 454.716;p < 0.001
Stroke	5	0.974	0.919	0.992	
Multiple sclerosis	2	0.816	0.217	0.969	
Spinal cord injury	2	0.965	0.793	0.995	
Cervical spondylotic myelopathy	1	0.800	−0.161	0.982	
Spinocerebellar ataxia	1	0.910	0.192	0.994	
Type 2 diabetes	2	0.880	0.443	0.979	
Chronic obstructive pulmonary disease	1	0.880	0.100	0.990	
End-stage renal disease	1	0.840	−0.039	0.986	
Total knee artroplasty	1	0.950	0.511	0.996	
Diverse neurological diagnoses	5	0.968	0.903	0.990	

K: number of studies; ICC_+ _: Mean intraclass correlation; LL and LU: lower and upper 95% confidence limits for ICC; F: Knapp-Hartung’s statistic for testing the significance of the moderator variable; Q_W_: statistic for testing the model misspecification; R^2^: proportion of variance accounted for by the moderator. The average reliability coefficients and their confidence limits were back-transformed to the original metric.

#### Brief-BESTest scale.

The simple regression meta-analyses of the continuous moderating variables for the alpha coefficient on the Brief-BESTest scale are shown in Table 13. In this case, only the mean age variables were marginally significant (p = 0.094; R^2^ = 39.2), with a positive regression weight, indicating an increase in the mean age of the sample led to an increase in the alpha coefficient. The rest of the moderators were not significant.

**Table 13:** Results of the simple meta-regression applied on alpha coefficient taking continuous moderator variables as predictors in the Brief-BESTest.

Predictor variable	K	b_j_	F	P	Q_E_	R^2^
Mean scores	3	−0.288	1.086	0.487	13.897***	1.03
SD of scores	3	0.817	11.025	0.186	2.546	92.4
Sample size	7	−0.002	0.009	0.928	49.770***	0
Mean age (years)	7	0.070	4.268	0.094	34.565***	39.2
SD of age (years)	6	−0.041	0.257	0.639	41.475***	0
Gender (% females)	7	0.016	0.886	0.390	43.749***	0
Year of study	7	0.042	0.155	0.710	47.891***	0
Experience with Brief-BESTest (years)	3	−0.031	0.019	0.912	26.188***	0

k: number of studies; b_j_: regression coefficient of each predictor; F: Knapp-Hartung’s statistics for testing the significance of the predictor (degree freedom 1 and k-2); p: probability level for the F statistic; Q_E_: statistics for testing the model misspecification; R^2^: proportion of variance accounted by the predictor. Regression coefficients were back-transformed to the original metric.

The weighted ANOVA of the categorical variables for the alpha coefficient on the Brief-BESTest scale is shown in Table 14. No categorical moderator was significant in explaining variation in the alpha coefficient.

**Table 14:** Results of the ANOVAs for the qualitative methodological and substantive moderator variables on alpha coefficient in the Brief-BESTest.

Variable	K	α	95% CI		ANOVA results
			LL	LU	
Test version					F(1,5) = 0.440;p = 0.537
Original	2	0.890	0.609	0.969	R^2^ = 0.0
Adaptation	5	0.924	0.844	0.963	Q_W_ = 48.286;p < 0.001
Type of design					F(1,5) = 0.440;p = 0.537
Observational	5	0.924	0.844	0.963	R^2^ = 0.0
Experimental	2	0.890	0.609	0.969	Q_W_ = 48.286;p < 0.001
Continent					F(2,4) = 0.375;p = 0.709
Asia	3	0.935	0.805	0.979	R^2^ = 0.0
Europe	2	0.906	0.658	0.974	Q_W_ = 41.902;p < 0.001
North America	2	0.890	0.531	0.974	
Population target 2					F(1,5) = 0.440;p = 0.537
Clinical	5	0.924	0.844	0.963	R^2^ = 0.0
Clinical + no-institucionalized	2	0.890	0.609	0.969	Q_W_ = 48.286;p < 0.001
Rater formation					F(1,5) = 2.329;p = 0.188
Physiotherapist	6	0.927	0.863	.952	R^2^ = 22.22
Other	1	0.818	0.241	0.956	Q_W_ = 37.643;p < 0.001
Disease					F(3,3) = 2.501;p = 0.236
Stroke	1	0.818	0.205	0.958	R^2^ = 44.51
Cervical spondylotic myelopathy	1	0.950	0.792	0.988	Q_W_ = 17.26;p = 0 < 0.001
Total knee arthropathy	2	0.948	0.860	0.981	
Combination	3	0.889	0.732	0.954	

K: number of studies; ICC_+ _: Mean coefficient alpha; LL and LU: lower and upper 95% confidence limits for α; F: Knapp-Hartung’s statistic for testing the significance of the moderator variable; Q_W_: statistic for testing the model misspecification; R^2^: proportion of variance accounted for by the moderator. The average reliability coefficients and their confidence limits were back-transformed to the original metric.

The simple meta-regressions of the continuous variables for ICCs (inter-rater agreement) in the Brief-BESTest are shown in Table 15. Mean scores were again significant (p = 0.005; R^2^ = 67.13) with a negative weight, indicating an increase in the group mean led to a decrease in inter-rater ICC. The rest of the continuous moderators were not significant.

**Table 15:** Results of the simple meta-regression applied on intraclass coefficient (interrater agreement) taking continuous moderator variables as predictors in the Brief-BESTest.

Predictor variable	K	b_j_	F	P	Q_E_	R^2^
Mean scores	10	−0.087	14.188	0.005	26.084**	67.13
SD of scores	10	0.042	0.266	0.620	65.987***	0
Sample size	13	−0.003	2.450	0.146	69.928***	13.74
Mean age (years)	13	−0.002	0.012	0.914	125.788***	0
SD of age (years)	12	−0.037	0.603	0.454	89.504***	0
Gender (% females)	11	0.001	0.016	0.902	123.008***	0
Year of study	13	−0.059	0.999	0.339	126.239***	0
Experience with Brief-BESTest (years)	6	−0.038	3.119	0.152	21.685***	28.44
Number of raters	13	0.119	0.465	0.510	119.662***	0
Sample size ICC (interrater)	13	0.008	0.416	0.532	87.328***	0

k: number of studies; b_j_: regression coefficient of each predictor; F: Knapp-Hartung’s statistics for testing the significance of the predictor (degree freedom 1 and k-2); p: probability level for the F statistic; Q_E_: statistics for testing the model misspecification; R^2^: proportion of variance accounted by the predictor. Regression coefficients were back-transformed to the original metric.

The weighted ANOVA of the categorical moderators in the ICC (inter-rater agreement) for the Brief-BESTest is shown in Table 16. Only continent the study proceeded from was marginally significant (p = 0.092; R^2^ = 50.38) with Australia showing lowest interrater agreement (ICC_+_=0.860) and with South America showing highest interrater agreement (ICC_+_=0.993).

**Table 16:** Results of the ANOVAs for the qualitative methodological and substantive moderator variables on intraclass coefficient (interrater agreement) in the Brief-BESTest.

Variable	K	ICC_+_	95% CI		ANOVA results
			LL	LU	
Test version					F(1,11) = 0.013;p = 0.917
Original	4	0.963	0.888	0.988	R^2^ = 0.0
Adaptation	9	0.965	0.931	0.983	Q_W_ = 126.729;p < 0.001
Type of design					F(1,11) = 2.382;p = 0.151
Observational	10	0.957	0.923	0.977	R^2^ = 9.48
Experimental	3	0.983	0.945	0.995	Q_W_ = 112.141;p < 0.001
Continent					F(4,8) = 2.917;p = 0.092
Asia	6	0.969	0.937	0.984	R^2^ = 50.38
Australia	1	0.860	0.399	0.974	Q_W_ = 35.360;p < 0.001
Europe	3	0.938	0.854	0.974	
North America	2	0.976	0.916	0.993	
South America	1	0.993	0.965	0.999	
Population target 2					F(2,10) = 2.333;p = 0.147
Clinical	11	0.962	0.934	0.978	R^2^ = 18.03
No-institucionalized	1	0.930	0.645	0.988	Q_W_ = 103.688;p < 0.001
Clinical + no-institucionalized	1	0.994	0.955	0.999	
Rater formation					F(3,9) = 0.110;p = 0.952
Physiotherapist	9	0.965	0.924	0.984	R^2^ = 0.0
Physiotherapist (no ended)	2	0.955	0.773	0.992	Q_W_ = 121.174;p < 0.001
Other	1	0.965	0.675	0.997	
Physiotherapist (ended + no ended)	1	0.980	0.727	0.999	
Disease					F(7,5) = 0.178;p = 0.979
No disease	2	0.977	0.786	0.998	R^2^ = 0.0
Parkinson´s disease	1	0.965	0.323	0.999	Q_W_ = 91.239;p < 0.001
Stroke	3	0.970	0.797	0.996	
Cervical spondylotic myelopathy	1	0.970	0.410	0.999	
Chronic obstructive pulmonary disease	2	0.935	0.467	0.994	
Total knee artroplasty	1	0.970	0.359	0.999	
Diverse neurological diagnoses	2	0.973	0.740	0.998	
Cancer	1	0.920	−0117	0.997	

K: number of studies; ICC_+ _: Mean intraclass correlation; LL and LU: lower and upper 95% confidence limits for ICC; F: Knapp-Hartung’s statistic for testing the significance of the moderator variable; Q_W_: statistic for testing the model misspecification; R^2^: proportion of variance accounted for by the moderator. The average reliability coefficients and their confidence limits were back-transformed to the original metric.

The simple meta-regressions of the continuous variables for the ICC (intra-rater agreement) of the Brief-BESTest are shown in Table 17. In this case, the number of raters was significant (p = 0.009; R^2^ = 39.84) with a positive regression weight (0.435), indicating an increase in the number of raters led to an increase in intra-rater ICC. The mean age of the sample was also significant (p = 0.032; R^2^ = 32.79) with a negative weight, indicating an increase in the mean age led to a decrease in intra-rater ICC.

**Table 17:** Results of the simple meta-regression applied on intraclass coefficient (intra-rater agreement) taking continuous moderator variables as predictors in the Brief-BESTest.

Predictor variable	K	b_j_	F	P	Q_E_	R^2^
Mean scores	12	−0.071	1.964	0.191	140.723***	9.10
SD of scores	12	−0.135	1.582	0.237	171.762***	2.76
Sample size	14	−0.002	0.417	0.531	178.826***	0
Mean age (years)	14	−0.053	5.892	0.032	115.940***	32.79
SD of age (years)	14	0.046	0.711	0.416	179.262***	0
Gender (% females)	12	−0.012	1.699	0.222	111.548***	8.06
Year of study	14	0.076	0.696	0.421	167.002***	0
Experience with Brief-BESTest (years)	6	−0.038	0.332	0.560	95.181***	0
Interval intra-rater	13	−0.014	0.309	0.590	172.395***	0
Number of raters	14	0.435	9.597	0.009	153.388***	39.84
Sample size ICC (intra-rater)	14	−0.006	0.113	0.743	176.737***	0

k: number of studies; b_j_: regression coefficient of each predictor; F: Knapp-Hartung’s statistics for testing the significance of the predictor (degree freedom 1 and k-2); p: probability level for the F statistic; Q_E_: statistics for testing the model misspecification; R^2^: proportion of variance accounted by the predictor. Regression coefficients were back-transformed to the original metric.

The weighted ANOVA of the categorical variables for the ICC (intra-rater agreement) in the Brief-BESTest is shown in Table 18. Design type was significant (p = 0.028; R^2^ = 28.17), with experimental studies showing a higher mean reliability (ICC_+_ = 0.991) than observational studies (ICC_+_ = 0.932). Rater formation was also significant (p = 0.051; R^2^ = 37.89) where the combination of raters with completed and unfinished physiotherapy studies obtained higher intra-rater agreement (ICC_+_ = 0.998) than physiotherapists with completed studies (ICC_+_ = 0.925) or only physiotherapists in training (ICC_+_ = 0.960).

**Table 18:** Results of the ANOVAs for the qualitative methodological and substantive moderator variables on intraclass coefficient (intra-rater agreement) in the Brief-BESTest.

Variable	K	ICC_+_	95% CI		ANOVA results
			LL	LU	
Test version					F(1,12) = 1.525;p = 0.240
Original	11	0.937	0.867	0.971	R^2^ = 1.52
Adaptation	3	0.976	0.890	0.995	Q_W_ = 177.064;p < 0.001
Type of design					F(1,12) = 6.264;p = 0.028
Observational	12	0.932	0.876	0.964	R^2^ = 28.17
Experimental	2	0.991	0.953	0.998	Q_W_ = 156.468;p < 0.001
Continent					F(4,9) = 1.393;p = 0.311
Asia	7	0.973	0.930	0.990	R^2^ = 17.36
Australia	1	0.930	0.325	0.995	Q_W_ = 95.598;p < 0.001
Europe	3	0.867	0.598	0.961	
North America	1	0.940	0.389	0.996	
South America	1	0.939	0.416	0.995	
Rater formation					F(3,10) = 3.699;p = 0.051
Physiotherapist	10	0.925	0.861	0.961	R^2^ = 37.89
Physiotherapist (no end)	2	0.960	0.831	0.991	Q_W_ = 135.424;p < 0.001
Other	1	0.973	0.803	0.997	
Physiotherapist (ended + no ended)	1	0.998	0.977	0.999	
Disease					F(9,4) = 1.106;p = 0.499
No disease	2	0.893	0.295	0.998	R^2^ = 9.67
Parkinson’s disease	1	0.973	0.482	0.999	Q_W_ = 30.712;p < 0.001
Stroke	3	0.986	0.902	0.998	
Cervical spondylotic myelopathy	1	0.990	0.780	0.999	
Spinocerebellar ataxia	1	0.810	−0.582	0.993	
Total knee arthropathy	1	0.940	0.113	0.998	
Cancer	1	0.940	0.070	0.998	
End-stage renal disease	1	0.840	−0.362	0.993	
Chronic obstructive pulmonary disease	2	0.886	0.237	0.998	
Diverse nuerological diagnoses	1	0.940	0.162	0.997	

K: number of studies; ICC_+ _: Mean intraclass correlation; LL and LU: lower and upper 95% confidence limits for ICC; F: Knapp-Hartung’s statistic for testing the significance of the moderator variable; Q_W_: statistic for testing the model misspecification; R^2^: proportion of variance accounted for by the moderator. The average reliability coefficients and their confidence limits were back-transformed to the original metric.

### Analysis of publication bias

The results of Egger’s test to examine publication bias in the eight meta-analyses in this study are shown in Table 19.

**Table 19:** Egger’s test results.

Scale	k	T	Df	P
BESTest				
Alpha	4	−1.573	2	0.116
ICCInterrater	19	−1.030	17	0.303
ICCIntrarater	24	1.714	22	0.086
MiniBESTest				
Alpha	24	−0.930	22	0.399
ICCInterrater	30	1.536	28	0.136
ICCIntrarater	33	0.530	31	0.600
Brief-BESTest				
Alpha	7	−0.141	5	0.894
ICCInterrater	13	1.297	11	0.221
ICCIntrarater	14	1.616	12	0.132

k = number of studies; t: test de Egger; df =  degree freedom; p =  probability value.

**Fig 10:**
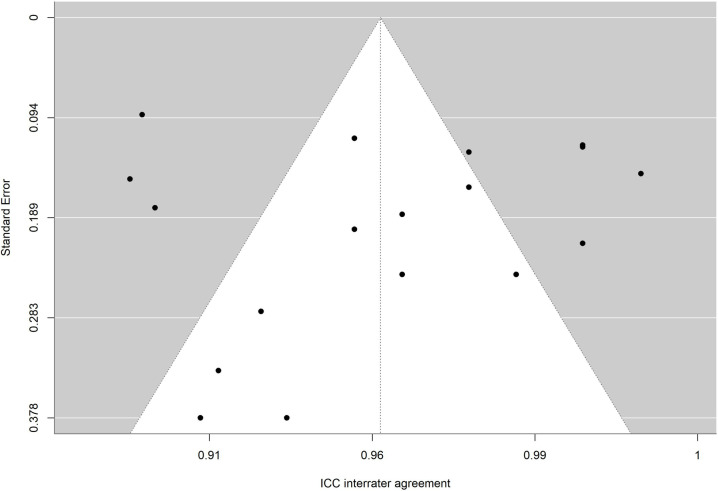
Funnel plot BESTest ICC inter-rater agreement.

**Fig 11:**
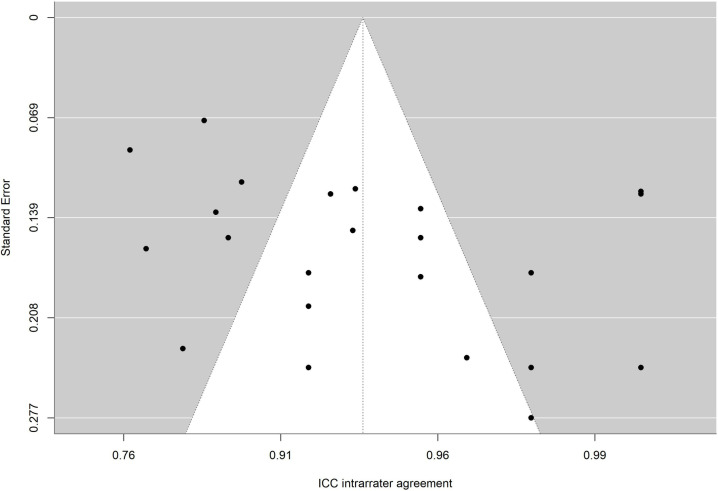
Funnel plot BESTest ICC intra-rater agreement.

**Fig 12:**
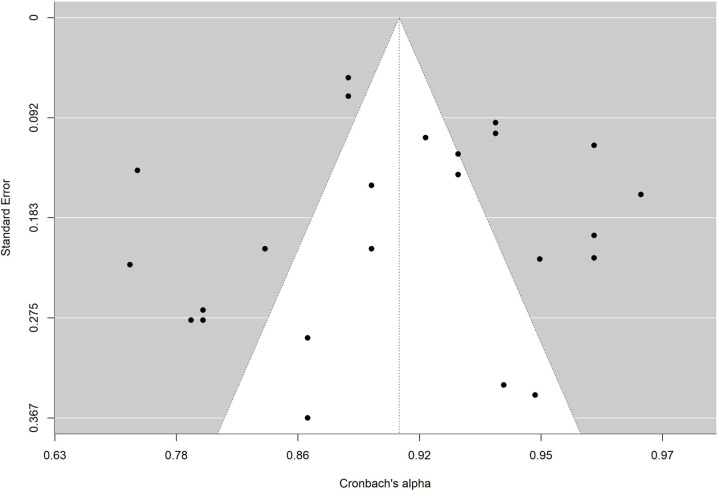
Funnel plot Mini-BESTest Cronbach´s alpha.

**Fig 13:**
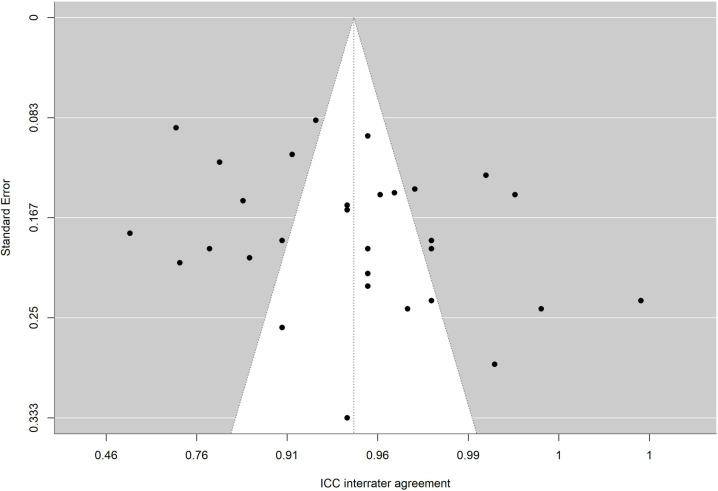
Funnel plot Mini-BESTest inter-rater agreement.

**Fig 14:**
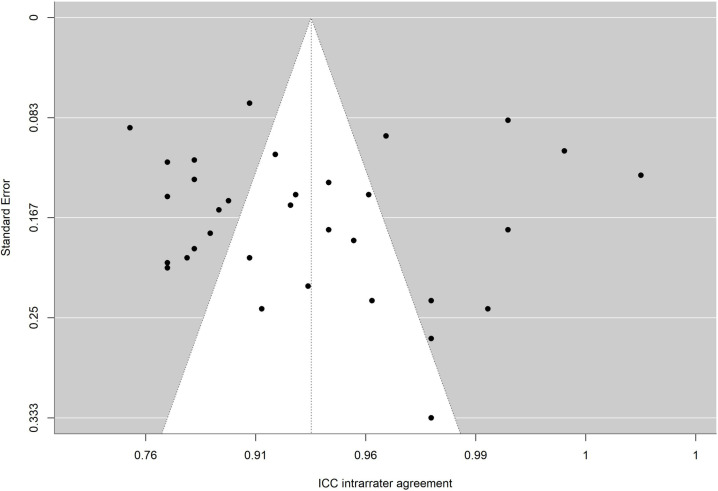
Funnel plot Mini-BESTest intra-rater agreement.

**Fig 15:**
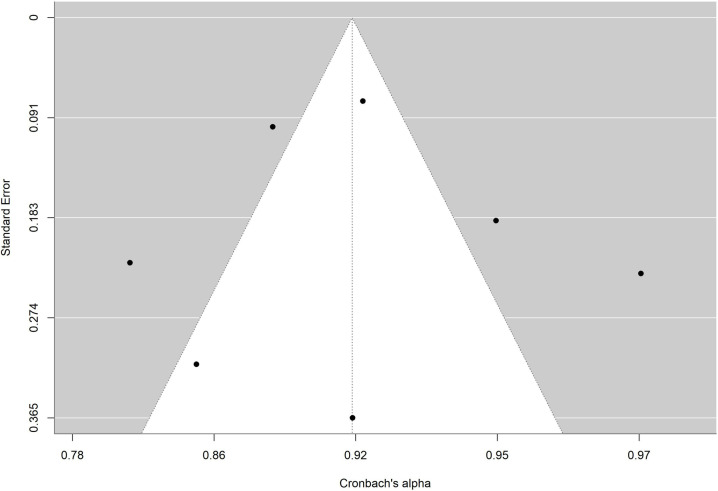
Funnel plot Brief-BESTest Cronbach´s alpha.

**Fig 16:**
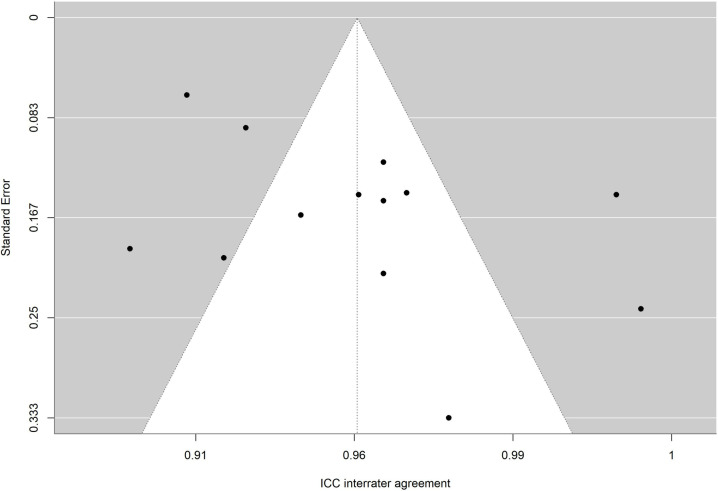
Funnel plot Brief-BESTest inter-rater agreement.

**Fig 17:**
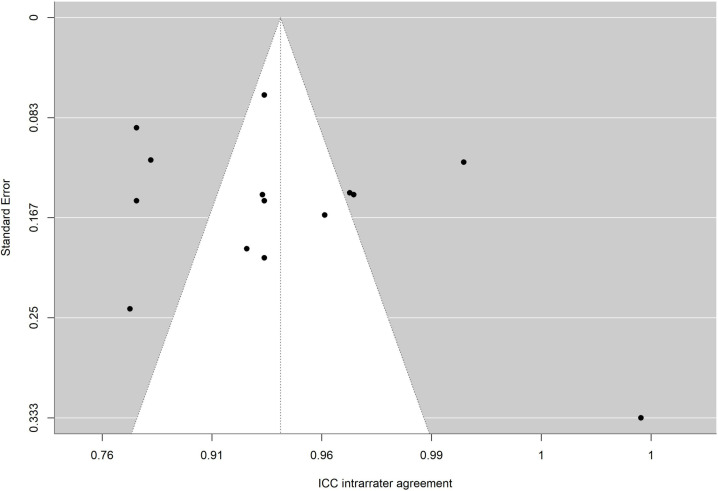
Funnel plot Brief-BESTest intra-rater agreement.

The absence of significance in Egger’s test rules out publication bias. In addition, the funnel plot is presented and the trim and fill method for imputing missing data [67] was applied. Figs 10,11,12,13,14,15,16,17 present funnel-plots of the mean reliability coefficients in the eight meta-analyses carried out with the BESTest, the Mini-BESTest and the Brief-BESTest, respectively. In no case is it observed that the trim and fill method has imputed data, thus publication bias is ruled out as a threat against results of meta-analyses.

## Discussion

We performed RG meta-analysis to determine how reliability of test scores varies in different test applications and which factors can explain that variability. This investigation is the first meta-analysis on the inter- and intra-rater (test-retest) reliability and internal consistency of the BESTest, Mini-BESTest and Brief-BESTest. This research is important as clinicians and researchers, to guide decision making, need outcome measures capable of accurately assessing balance control in patients with neurological pathology, those with musculoskeletal problems, older adults and children without pathology, and patients with other pathologies.

Regarding Intraclass Correlation Coefficient (ICC) for reliability, Roach and Toomey and Coote [96,97] showed ICC values over 0.75 as excellent, 0.40–0.75 as moderate and below 0.4 as poor reliability, and Munro et al. [98] proposed interpreting the clinical significance of ICC following this guide (acceptable alpha above 0.7, at values between 0.7–0.8 as good and at values above 0.8 as excellent). The mean intraclass correlations and Cronbach alpha obtained for BESTest, Mini-BESTest and Brief-BESTest in our meta-analysis exhibited excellent inter and intra-rater reliability (ICC = 0.94–0.97) and internal consistency (alpha = 0.92). Considering the guidelines of Munro et al. [98], the average reliability obtained in this study make three scales adequate to be applied to different populations for screening balance problems in different populations.

The methodological quality of most of the included studies of the three scales was very good and sufficient for internal consistency and doubtful and sufficient for inter-rater and intra-rater/test-retest reliability. Most obtained doubtful methodological quality as they did not indicate whether patients were stable or if test conditions were similar. Studies should provide any evidence that patients were stable to increase the methodological quality of studies for inter-rater and intra-rater/test-retest. Another aspect to consider when assessing the test-retest or intra-rater reliability of the test is an adequate time interval between both test administrations. This should be short enough to avoid significant changes in the patient’s condition and long enough to avoid recall bias.

Large heterogeneity among coefficients was found for BESTest, Mini-BESTest and Brief-BESTest, therefore we performed moderator analyses to identify which study characteristics could explain this variability. For continuous moderators, we found that mean scores were statistically associated with inter-rater reliability, and that mean of disorder history had marginal statistical significance for the intra-rater reliability of BESTest. As the mean of the scale scores increases, interrater reliability decreases. It seems that the higher the score on the BESTest scale, the lower the inter-rater reliability.

As regards the Mini-BESTest, the raters´ experience was statistically associated with inter-rater reliability. Thus, as the experience of raters increases, inter-rater reliability decreases. This may be because less experienced raters are more meticulous and rigorous in applying and evaluating the scales. Furthermore, the mean history of the disorder was significant for intra-rater reliability, indicating an increase in number of years with the disorder suffered by patients implied a decrease in intra-rater agreement. This may be because as a patient with a neurological or musculoskeletal pathology becomes chronic, they adopt a series of compensations that may influence assessment of balance control. Standard deviation of scores and gender were marginally and significant statistically associated with internal consistency, respectively. An increase in standard deviation of scores and a decrease in the number of women in the study sample implies an increase in internal consistency of the Mini-BESTest. Although standard deviation of test scores explains an important part of variance, this did not reach statistical significance. This lack of statistical significance could be due to low statistical power. Standard deviation of scores has previously been found to be a source of systematic variation of reliability coefficients [99]. Psychometric theory states the higher the SD of test scores, the higher reliability obtained [51].

As for the Brief-BESTest, mean scores, number of raters and mean age of sample were statistically associated with inter-rater and intra-rater reliability respectively, so as the average of scale scores increases and number of raters decreases interrater reliability and increases intra-rater reliability. The latter appears to be higher when several raters rather than a single rater administer the scale to patients on two different occasions. Furthermore, it appears that as the age of the sample increases, intra-rater reliability decreases. The mean age of the sample was also marginally significant indicating an increase in the mean age led to an increase in internal consistency.

As regards the qualitative moderator analysis (ANOVAs), we found that, in the BESTest, the continent the study proceeded from was a significant moderator for inter-rater reliability. The lowest inter-rater ICC was obtained in Australia the highest (on average) in Asia. Furthemore, the continent the study proceeded from obtained marginal statistical significance in the intra-class intra-rater correlation of BESTest and inter-rater correlation of Mini-BESTest. The disease and population type were marginally significant and significant moderators for internal consistency and inter-rater reliability of Mini-BESTest, respectively. In relation to disease, the lowest coefficient was obtained in patients with type 2 diabetes and the highest in patients with total knee arthroplasty. Balance problems may be more readily observed when assessed in patients suffering from a musculoskeletal problem associated with surgery than when patients have neuropathic involvement associated with a metabolic problem such as diabetes. Also the population type was significant with the normal institutionalized population indicating a higher inter-rater reliability than the mixed population or clinical population. The lowest coefficient was obtained in the normal, non-institutionalized population.

For the Brief-BESTest, the type of design was significant where experimental studies showed a higher mean intra-rater reliability than observation studies. An explanation is the number of experimental studies was significantly lower than that of observational studies and that in the former, evaluations can be conducted by expert judges. Rater formation was also significant where the combination of raters with completed and unfinished physiotherapy studies obtained higher intra-rater agreement than physiotherapists with completed studies or only physiotherapists in training.

### Limitations

Our study has several limitations. The number of studies reporting reliability estimates with data at hand is considerably smaller for BESTest and especially for the Brief-BESTest. This, together with the lack of important data reported by authors reduced the possibility of analyzing their influence as potential moderating variables on reliability coefficients. In particular, many studies did not report the mean and standard deviation of disorder and experience with the scale (BESTest, Mini-BESTest or Brief-BESTest). Furthermore, some studies did not report the mean and standard deviation of test scores, two essential moderators in the context of RG studies.

## Conclusions

The main findings of the current RG meta-analysis report that the BESTest, Mini-BESTest and Brief-BESTest instruments present, on average, excellent reliability and internal consistency values. These outcome measures can be recommended for the screening of balance control and balance impairments. Some continuous and categorical moderator variables increase reliability and internal consistency of these scales. Mean scores, standard deviation of scores, mean age, gender, population type, mean history of the disorder, disease, raters´ experience, number of raters, rater formation, continent of study and design type presented statistically significant relationships with ICC and/or Cronbach´s alpha for BESTest and the two abbreviated versions.

## Supporting information

S1 Table Search Strategy. (DOCX)

S2 Table Studies included and excluded. (XLSX)

S3 Table Checklist PRISMA. (DOCX)

S4 Table Characteristics of the included studies. (DOCX)

S5 Table Evaluating of methodological quality. (DOCX)
