# Supplementary material for: The balance evaluation systems test (BESTest), mini-BESTest and brief-BESTest as clinical tools to assess balance control across different populations: A reliability generalization meta-analysis
Source: PLoS One. 2025 Apr 3;20(4):e0318302. doi: 10.1371/journal.pone.0318302 (PMC11967966; doi:10.1371/journal.pone.0318302)
Supplement: S1 Table — (DOCX) [file pone.0318302.s001.docx]

S1 Table. Search Strategy

| Database | Search Strategy |
| --- | --- |
| PubMed | ("BESTest" OR "balance evaluation systems test" OR "Mini-BESTest" OR "Mini-balance evaluation systems test" OR "Brief-BESTest" OR “Brief-balance evaluation systems test”) AND (“internal consistency” OR reliability OR “test-retest reliability” OR “inter-rater reliability” OR “interrater reliability” OR “intra-rater reliability” OR “intrarater reliability”)  In: Title/abstact |
| Embase | ("BESTest" OR "balance evaluation systems test" OR "Mini-BESTest" OR "Mini-balance evaluation systems test" OR "Brief-BESTest" OR “Brief-balance evaluation systems test”) AND (“internal consistency” OR reliability OR “test-retest reliability” OR “inter-rater reliability” OR “interrater reliability” OR “intra-rater reliability” OR “intrarater reliability”)  In: Title/abstact/key words |
| WoS | ("BESTest" OR "balance evaluation systems test" OR "Mini-BESTest" OR "Mini-balance evaluation systems test" OR "Brief-BESTest" OR “Brief-balance evaluation systems test”) AND (“internal consistency” OR reliability OR “test-retest reliability” OR “inter-rater reliability” OR “interrater reliability” OR “intra-rater reliability” OR “intrarater reliability”)  In: Topic |
| Scopus | ("BESTest" OR "balance evaluation systems test" OR "Mini-BESTest" OR "Mini-balance evaluation systems test" OR "Brief-BESTest" OR “Brief-balance evaluation systems test”) AND (“internal consistency” OR reliability OR “test-retest reliability” OR “inter-rater reliability” OR “interrater reliability” OR “intra-rater reliability” OR “intrarater reliability”)  In: Topic |
| PsycINFO | ("BESTest" OR "balance evaluation systems test" OR "Mini-BESTest" OR "Mini-balance evaluation systems test" OR "Brief-BESTest" OR “Brief-balance evaluation systems test”) AND (“internal consistency” OR reliability OR “test-retest reliability” OR “inter-rater reliability” OR “interrater reliability” OR “intra-rater reliability” OR “intrarater reliability”)  In: TX full text |
| CINAHL | ("BESTest" OR "balance evaluation systems test" OR "Mini-BESTest" OR "Mini-balance evaluation systems test" OR "Brief-BESTest" OR “Brief-balance evaluation systems test”) AND (“internal consistency” OR reliability OR “test-retest reliability” OR “inter-rater reliability” OR “interrater reliability” OR “intra-rater reliability” OR “intrarater reliability”)  In: TX full text |
