# Supplementary material for: The balance evaluation systems test (BESTest), mini-BESTest and brief-BESTest as clinical tools to assess balance control across different populations: A reliability generalization meta-analysis
Source: PLoS One. 2025 Apr 3;20(4):e0318302. doi: 10.1371/journal.pone.0318302 (PMC11967966; doi:10.1371/journal.pone.0318302)
Supplement: S4 Table — (DOCX) [file pone.0318302.s004.docx]

S4 Table. Characteristics of the included studies (all studies are eligible to be included in the systematic review)

| **Study**  **Year**  **Country**  **Name of data extractors/date of data extraction** | **Sample/s** | **BESTest, Mini-BESTest and/or Brief-BESTest**  **(mean/ SD)** | **Population** | **Psychometric study**  **(BESTest, Mini-BESTest, Brief-BESTest/and other/s scale/s)** | **Rater/s** | **Type/s of reliability** | **Time interval**  **(Inter-rater and/or intra-rater reliability)** |  |
| --- | --- | --- | --- | --- | --- | --- | --- | --- |
| Horak FB et al. 2009 [1]  USA  ABMH and JALP  (4 March-19 April 2024) | N=12  Age (mean/SD): 63.0/10.0  Gender (% male): 41.67  Disease: Parkinson disease, vestibular dysfunction, peripheral neuropathy, total hipo artroplasty  Disorder history (mean/SD): 0.33y/? | BESTest:45.04/? | Communitary normal, clinical | BESTest: Yes  Other scale: No | Number: 9 inter-rater  Profession: physical therapist  Experience (Yes or No/ time):?/? | ICC inter-rater:0.91 | NA |  |
| Marques A et al.  2016 [13]  Portugal  ABMH and JALP  (4 March-19 April 2024) | N=122  Age (mean/SD):75.9/8.9  Gender (% male): 70.5  Disease: No  Disorder history (mean/SD):NA | BESTest:84.7/24.5  Mini-BESTest: 19.8/6.8  Brief-BESTest:14.3/6.8 | Normal institutionalised | BESTest: Yes  Mini-BESTest:Yes  Brief-BESTest:Yes  Other scale: No | Number: 2 inter-rater; 1 test-retest  Profession: physical therapist  Experience: (Yes or No/time):Yes/5y | BESTest  ICC inter-rater:0.86  ICC test-retest:0.77  Mini-BESTest  ICC inter-rater:0.71  ICC test-retest:0.73  Brief-BESTest  ICC inter-rater:0.93  ICC test-retest:0.82 | 2.5d test-retest |  |
| Nakhostin-Ansari A et al.  2022 [15]  Iran  ABMH and JALP  (4 March-19 April 2024) | N=49  Age (mean/SD): 60.8/13.9  Gender (% male): 32.7  Disease: parkinson disease  Disorder history (mean/SD):5.27y/4.62 | Mini-BESTest:21.2/3.88  Brief-BESTest:14.99/4.89 | Clinical | Mini-BESTest:Yes  Brief-BESTest:Yes  Other scale: No | Number: 3 inter-rater; 1 intra-rater  Profession: medical student  Experience (Yes or No/time):?/? | Mini-BESTest  ICC inter-rater:0.965  ICC intra-rater:0.965  Brief-BESTest  ICC inter-rater:0.965  ICC intra-rater:0.973 | 14d intra-rater |  |
| Continued. Table 2. | | | | | | | |  |
| Wallén MB et al.  2016 [17]  Sweden  ABMH and JALP  (4 March-19 April 2024) | N=112  Age (mean/SD):72.8/5.5  Gender (% male): 43  Disease: parkinson disease  Disorder history (mean/SD):?/? | Mini-BESTest:19.2/3.6 | Clinical | Mini-BESTest: Yes  Other scale: No | Number: NA  Profession: NA  -Physical therapist  -Occupational therapist  Experience (Yes or No/time):NA | α de Cronbach:0.738 | NA |  |
| Godi M et al.  2021[18]  Italy  ABMH and JALP  (4 March-19 April 2024) | N=709  Age (mean/SD):70.3/7.9  Gender (% male): 41.61  Disease: Parkinson disease  Disorder history (mean/SD):?/? | Mini-BESTest:?/? | Clinical | Mini-BESTest: Yes  Other scale: No | Number: NA  Profession:?  Experience (Yes or No/time):?/? | α de Cronbach:0.89 | NA |  |
| Yingyongyudha A et al.  2016 [19]  Thailand  ABMH and JALP  (4 March-19 April 2024) | N=200  Age (mean/SD):70.25/7.05  Gender (% male): 63  Disease: No  Disorder history (mean/SD):NA | BESTest: 66.11/9.89  Mini-BESTest:15.9/5.80 | Community normal | BESTest: Yes  Mini-BESTest:Yes  Other scale: No | Number: 1 intra-rater  Profession:?  Experience (Yes or No/time):?/? | BESTest  ICC intra-rater:0.85  Mini-BESTest  ICC intra-rater:0.90 | ? |  |
| Phyu SN et al.  2022a [20]  Thailand  ABMH and JALP  (4 March-19 April 2024) | N=44  Age (mean/SD): 56.61/7.70  Gender (% male): 90.9  Disease: type 2 diabetes with peripheral neuropathy  Disorder history (mean/SD):8.43y/3.30 | Mini-BESTest:21.4/2.61 | Clinical | Mini-BESTest: Yes  Other scale: No | Number: 2 inter-rater; 1 intra-rater  Profession: Physical therapist  Experience (Yes or No/time): Yes/11.5y | α de Cronbach:0.73  ICC inter-rater:0.95  ICC intra-rater:0.93 | 8.5d intra-rater |  |
| Continued. Table 2. | | | | | | | |  |
| Phyu SN et al.  2022b [21]  Thailand  ABMH and JALP  (4 March-19 April 2024) | N=48  Age (mean/SD): 59.04/7.53  Gender (% male): 93.75  Disease: type 2 diabetes with peripheral neuropathy  Disorder history (mean/SD):10.58y/3.76 | Mini-BESTest:17.02/1.64 | Clinical | Mini-BESTest: Yes  Other scale:No | Number: 1 intra-rater  Profession: Physical therapist  Experience (Yes or No/time):Yes/25y | ICC intra-rater:0.80 | ? |  |
| Chinsongkram B et al.  2014 [22]  Thailand  ABMH and JALP  (4 March-19 April 2024) | N=70  Age (mean/SD): 57.01/12.23  Gender (% male): 45.71  Disease: stroke  Disorder history (mean/SD): 0.33y/? | BESTest:45.04/? | Clinical | BESTest: Yes  Other scale: No | Number: 5 inter-rater, 1 intra-rater  Profession: physical therapist  Experience (Yes or No/ time):Yes/5.33y | ICC inter-rater:0.99  ICC intra-rater:0.99 | 7d intra-rater |  |
| Madsalae T et al.  2022 [23]  Thailand  ABMH and JALP  (4 March-19 April 2024) | | N=60  Age (mean/SD): 64.70/3.74  Gender (% male): 76.67  Disease: chronic neck pain  Disorder history (mean/SD): ?/? | BESTest:?/? | Communitary normal | BESTest: Yes  Other scale: No | Number: 1 intra-rater  Profession: ?  Experience (Yes or No/ time):?/? | ICC intra-rater:0.96 | ? |
| Rodrigues LC et al.  2014 [24]  Brazil  ABMH and JALP  (4 March-19 April 2024) | N=16  Age (mean/SD): 61.1/7.5  Gender (% male): 18.75  Disease: stroke  Disorder history (mean/SD): 13.38y/9.6 | BESTest:?/? | Clinical | BESTest: Yes  Other scale: No | Number: 2 inter-rater,1 test-retest  Profession: physical therapist  Experience (Yes or No/ time):?/? | BESTest  ICC inter-rater:0.93  ICC test-retest:0.98 | 7d test-retest |  |
| Continued. Table 2. | | | | | | | |  |
| Pereira Viveiro LA et al.  2019 [25]  Brazil  ABMH and JALP  (4 March-19 April 2024) | N=49  Age (mean/SD): 77.8/7.2  Gender (% male): 61.2  Disease: No  Disorder history (mean/SD):NA | BESTest: 53.2/24.2  Mini-BESTest:  42.12/10.39  Brief-BESTest:7.5/5.8 | Institutionalised normal | BESTest:Yes  Mini-BESTest:Yes  Brief-BESTest:Yes  Other scale: No | Number: 2 inter-rater; 1 test-retest  Profession: Physical therapist  Experience (Yes or No/time):Yes/? | BESTest  ICC inter-rater:0.993  ICC test-retest: 0.939  Mini-BESTest  ICC inter-rater: 0.992  ICC test-retest:0.933  Brief-BESTest  ICC inter-rater: 0.993  ICC test-retest:0.939 | 10.5d test-retest |  |
| Maia AC et al.  2013 [26]  Brazil  ABMH and JALP  (4 March-19 April 2024) | Sample a (Maia AC et al, 2013a)  N=35  Age (mean/SD):66.5/10.3  Gender (% male):40  Disease: Parkinson disease  Disorder history (mean/SD):9.9y/6.4 | BESTest:?/?  Mini-BESTest:? | Clinical | BESTest: Yes  Mini-BESTest:Yes  Other scale: No | Number: 1 intra-rater  Profession:?  Experience (Yes or No/time):?/? | BESTest  ICC intra-rater:0.92  Mini-BESTest  ICC intra-rater:0.95 | 7d intra-rater |  |
|  | Sample b (Maia AC et al, 2013b)  N=35  Age (mean/SD):73.8/7.4  Gender (% male):74.29  Disease: No  Disorder history (mean/SD):NA | BESTest:?/?  Mini-BESTest:? | Normal institutionalised | BESTest: Yes  Mini-BESTest:Yes  Other scale: No | Number: 1 intra-rater  Profession:?  Experience (Yes or No/time):?/? | BESTest  ICC intra-rater:0.98  Mini-BESTest  ICC intra-rater:0.99 | 7d intra-rater |  |
| Jácome C et al.  2016 [27]  Portugal  ABMH and JALP  (4 March-19 April 2024) | N=46  Age (mean/SD):75.9/7.1  Gender (% male): 47.8  Disease: chronic obstructive pulmonary disease  Disorder history (mean/SD):?/? | BESTest: 77.8/12.5  Mini-BESTest:20.8/4.9  Brief-BESTest:15.7/4.9 | Clinical | BESTest: Yes  Mini-BESTest:Yes  Brief-BESTest:Yes  Other scale: No | Number: 2 inter-rater, 1 intra-rater  Profession: Physical therapist  Experience (Yes or No/time):Yes/4y | BESTest  ICC inter-rater:0.85  ICC intra-rater:0.87  Mini-BESTest  ICC inter-rater:0.85  ICC intra-rater: 0.88 | 2.5d intra-rater |  |
|  |  |  |  |  |  | Brief-BESTest  ICC inter-rater:0.97  ICC test-retest:0.82 |  |  |
| Continued. Table 2. | | | | | | | |  |
| Jácome C et al.  2018 [28]  Portugal  ABMH and JALP  (4 March-19 April 2024) | N=74  Age (mean/SD): 63.9/15.1  Gender (% male): 33.8  Disease: end-stage renal disease  Disorder history (mean/SD):21y/15 | BESTest:77.9/17.3  Mini-BESTest:21.7/6.0  Brief-BESTest:14.9/6.4 | Clinical | BESTest:Yes  Mini-BESTest.Yes  Brief-BESTest:Yes  Other scale: No | Number: 2 inter-rater, 1 test-retest  Profession: Physical therapist  Experience: (Yes or No/time):?/? | BESTest  ICC test-retest:0.94  Mini-BESTest  ICC test-retest:0.84  Brief-BESTest  ICC test-retest:0.84 | 5d intra-rater |  |
| Naghdi S et al.  2020 [29]  Iran  ABMH and JALP  (4 March-19 April 2024) | N=30  Age (mean/SD):54.2/16.1  Gender (% male): 43.3  Disease: stroke  Disorder history (mean/SD):? | Mini-BESTest:19.55/5.45 | Clinical/  Institutionalized | Mini-BESTest:Yes  Other scale: NA | Number: 1  Profession: Physical therapist/doctor  Experience (Yes or No/time):Yes/? | ICC inter-rater:0.98 | NA |  |
| Bahirei S et al.  2023 [30]  Iran  ABMH and JALP  (4 March-19 April 2024) | N=65  Age (mean/SD): 21.20/4.80  Gender (% male): 39  Disease: intellectual disability  Disorder history (mean/SD): ? | BESTest:70.64/65.0 | Clinical | BESTest: Yes  Other scale: No | Number:?  Profession:?  Experience (Yes or No/ time):?/? | ICC test-retest:0.92 | ? |  |
| Lemay JF et al.  2019 [31]  Canada  ABMH and JALP  (4 March-19 April 2024) | N=20  Age (mean/SD):56.4/17.14  Gender (% male):40  Disease: spinal cord injury; stroke; orthopedic issue; TBI; spinal cord injury+cerebral palsy  Disorder history (mean/SD):? | Mini-BESTest:? | Clinical | Mini-BESTest:Yes  Other scale:No | Number: 2 inter-rater; 1 intra-rater  Profession: physical therpay  Experience (Yes or No/time):Yes/15y | ICC inter-rater:0.974  ICC test-retest:0.988 | 21d intra-rater |  |
| Continued. Table 2. | | | | | | | |  |
| Severijns P et al.  2019 [32]  Belgium  ABMH and JALP  (4 March-19 April 2024) | Sample a (Severijns P et al, 2019a)  N=10  Age (mean/SD): 59.6/8.3  Gender (% male): 70  Disease: spinal deformity  Disorder history (mean/SD): ?/? | BESTest:?/? | Clinical | BESTest: Yes  Other scale: No | Number: 3 inter-rater  Profession: physical therapist  Experience (Yes or No/ time):?/? | BESTest  ICC inter-rater:0.94 | NA |  |
|  | Sample b (Severijns P et al, 2019b)  N=10  Age (mean/SD): 63.0/13.30  Gender (% male): 70  Disease: spinal deformity  Disorder history (mean/SD): ?/? | BETest:?/? | Clinical | BESTest: Yes  Other scale: No | Number: 1 test-retest  Profession: physical therapist  Experience (Yes or No/ time):?/? | BESTest  ICC inter-rater:0.90  α de Cronbach:0.70 | 14d test-retest |  |
| Lampropoulou SI et al.  2019 [33]  Greece  ABMH and JALP  (4 March-19 April 2024) | N=21  Age (mean/SD):63.0/16.0  Gender (% male): 33.33  Disease: stroke  Disorder history (mean/SD): ? | Mini-BESTest:16.0/8.0 | Clinical | Mini-BESTest:Yes  Other scale: No | Number:2 inter-rater; 1 intra-rater  Profession: physical therapist  Experience (Yes or No / time): Yes/0.167y | α de Cronbach:0.942  ICC inter-rater:0.998  ICC test-retest:0.966 | 8.5d test-retest |  |
| Kondo Y et al.  2020 [34]  Japan  ABMH and JALP  (4 March-19 April 2024) | N=20  Age (mean/SD): 63.7/10.1  Gender (% male): 35  Disease: spinocerebellar ataxia  Disorder history (mean/SD): ? | BESTest:69.85/10.45  Mini-BESTest:?/?  Brief-BESTest:9.14/3.31 | Clinical | BESTest: Yes  Mini-BESTest:Yes Brief-BESTest:Yes Other scale:No | Number: 1 test-retest  Profession: Physical therapist  Experience (Yes or No/ time):Yes/? | BESTest  ICC test-retest:0.92  Mini-BESTest  ICC test-retest:0.91  Brief-BESTest  ICC test-retest:0.81 | 30d intra-rater |  |
| Continued. Table 2. | | | | | | | |  |
| Hamre Ch et al.  2017 [35]  Norway  ABMH and JALP  (4 March-19 April 2024) | N=42  Age (mean/SD):71.7/14.8  Gender (% male): 64.3  Disease: stroke, multiple sclerosis  Disorder history (mean/SD):?/? | BESTest: 80.55/15.23  Mini-BESTest:18.3/5.31 | Communitary normal, clinical | BESTest: Yes  Mini-BESTest:Yes  Other scale: No | Number: 1 test-retest  Profession: Physical therapist  Experience (Yes or No/time):Yes/18y | BESTest  ICC inter-rater:0.98  ICC test-retest:1.79  Mini-BESTest  ICC inter-rater:0.95  ICC intra-rater:0.87 | 2d |  |
| Goljar N et al.  2017 [36]  Slovenia, Italy, Croatia  ABMH and JALP  (4 March-19 April 2024) | N=159  Age (mean/SD):69.3/10.3  Gender (% male): 37.11  Disease: stroke  Disorder history (mean/SD):?/? | Mini-BESTest:?/? | Clinical | Mini-BESTest: Yes  Other scale: No | Number: NA  Profession:?  Experience (Yes or No/time):?/? | α de Cronbach:0.96 | NA |  |
| Aydogan Arslan SA et al.  2021 [37]  Turkey  ABMH and JALP  (4 March-19 April 2024) | N=40  Age (mean/SD): 60.28/9.96  Gender (% male): 27.5  Disease: stroke  Disorder history (mean/SD):? | Brief-BESTest:10.08/6.15 | Clinical | Brief-BESTest:Yes  Other scale: No | Number: 2 inter-rater, 1 intra-rater  Profession: Physical therapist  Experience: (Yes or No/time):Yes/? | ICC inter-rater:0.95  ICC intra-rater:0.965 | 7d intra-rater |  |
| Göktas A et al.  2020 [38]  Turkey  ABMH and JALP  (4 March-19 April 2024) | | N=84  Age (mean/SD):59.52/14.04  Gender (% male): 33.3  Disease: stroke  Disorder history (mean/SD):? | Mini-BESTest:9.21/8.74 | Clinical | Mini-BESTest: Yes  Other scale: No | Number: 2 test-retest  Profession: ?  Experience (Yes or No/time):?/? | ICC test-retest:0.994 | 7d test-retest |
| Continued. Table 2. | | | | | | | |  |
| Dogrouz Karatekin BD et al.  2023 [39]  Turkey  ABMH and JALP  (4 March-19 April 2024) | N=61  Age (mean/SD):61.72/8.58  Gender (% male): 44.3  Disease: Parkinson disease, stroke, multiple sclerosis.  Disorder history (mean/SD):5.62/3.97 | Mini-BESTest:15.03/7.02 | Clinical | Mini-BESTest: Yes  Other scale:No | Number:2 inter-rater, 1 test-retest  Profession: Physical Therapist  Experience (Yes or No/time):?/? | ICC inter-rater:0.989  ICC test-retest: 0.997 | 5d test-retest |  |
| Dewar R et al.  2017 [40]  Australia  ABMH and JALP  (4 March-19 April 2024) | Sample a (Dewar R et al, 2017a)  N=34  Age (mean/SD):10.8/2.2  Gender (% male): 44  Disease: No  Disorder history (mean/SD):NA | BESTest:?/?  Mini-BESTest:?/? | Community normal | BESTest: Yes  Mini-BESTest:Yes  Other scale:No | Number:2 inter-rater, 1 test-retest  Profession: physical therapist  Experience (Yes or No/time):Yes/19.5y | BESTest  ICC inter-rater:0.87  ICC test-retest:0.96  Mini-BESTest  ICC inter-rater:0.56  ICC test-retest:0.86 | 28d test-retest |  |
|  | Sample b (Dewar R et al, 2017b)  N=22  Age (mean/SD):10.9/1.6  Gender (% male): 55  Disease: No  Disorder history (mean/SD):NA | BESTest:?/?  Mini-BESTest:?/? | Community normal | BESTest: Yes  Mini-BESTest:Yes  Other scale:No | Number:1 test-retest  Profession: physical therapist  Experience (Yes or No/time):Yes/19.5y | BESTest  ICC test-retest:0.83  Mini-BESTest  ICC test-retest:? | 28d test-retest |  |
| Huang MH et al.  2016 [41]  USA  ABMH and JALP  (4 March-19 April 2024) | N=28  Age (mean/SD):68.4/8.13  Gender (% male): ?  Disease: cancer  Disorder history (mean/SD):6y/3.45 | BESTest: 90.0/7.4  Mini-BESTest:22.0/2.99  Brief-BESTest:16.8/3.48 | Clinical | BESTest: Yes  Mini-BESTest:Yes  Brief-BESTest:Yes  Other scale: No | Number: 2 inter-rater, 1 test-retest  Profession: Physical therapist  Experience (Yes or No/time):Yes/20y | BESTest  ICC inter-rater:0.96  ICC test-retest:0.92  Mini-BESTest  ICC inter-rater:0.86  ICC test-retest:0.90  Brief-BESTest  ICC inter-rater:0.92  ICC test-retest:0.94 | 10.5d test-retest |  |
| Continued. Table 2. | | | | | | | |  |
| Chan ACM et al.  2015 [42]  China  ABMH and JALP  (4 March-19 April 2024) | Sample a (Chan ACM et al, 2015a)  N=25  Age (mean/SD):69.7/6.8  Gender (% male): 68  Disease: total knee artroplasty  Disorder history (mean/SD):?/? | BESTest:?/?  Mini-BESTest:?/?  Brief-BESTest:?/? | Clinical | BESTest: Yes  Mini-BESTest:Yes  Brief-BESTest:Yes  Other scale: No | Number: 3 inter-rater  Profession: Physical therapist  Experience: (Yes or No/time):Yes/10y | BESTest  ICC inter-rater:0.99  α de Cronbach:0.98  Mini-BESTest  ICC inter-rater:0.96  Brief-BESTest  ICC inter-rater:0.97 | NA |  |
|  | Sample b (Chan ACM et al, 2015b)  N=46  Age (mean/SD):69.1/6.1  Gender (% male): 74  Disease: total knee artroplasty  Disorder history (mean/SD):?/? | BESTest:?/?  Mini-BESTest:?/?  Brief-BESTest:?/? | Clinical | BESTest: Yes  Mini-BESTest:Yes  Brief-BESTest:Yes  Other scale: No | Number: 1 intra-rater  Profession: Physical therapist  Experience: (Yes or No/time):Yes/10y | BESTest  ICC intra-rater:0.96  Mini-BESTest  ICC inter-rater:0.96  α de Cronbach:0.96  Brief-BESTest  ICC intra-rater:0.94  α de Cronbach:0.97 | 7d intra-rater |  |
| Chiu AYY et al.  2018 [43]  China  ABMH and JALP  (4 March-19 April 2024) | N=72  Age (mean/SD):63.9/10.9  Gender (% male): 31.94  Disease: cervical spondylotic myelopathy  Disorder history (mean/SD):?/? | BESTest:68.1/29.4  Mini-BESTest: 15.5/9.3  Brief-BESTest:10.1/6.6 | Clinical | BESTest:Yes  Mini-BESTest:Yes  Brief-BESTest:Yes  Other scale:No | Number:2 inter-rater, 1 test-retest  Profession: physical therapist  Experience (Yes or No/time):Yes/5y | BESTest  ICC inter-rater:0.99  ICC test-retest:0.99  Mini-BESTest  ICC inter-rater:0.81  ICC test-retest:0.80  Brief-BESTest  ICC inter-rater:0.97  ICC test-retest:0.99  α de Cronbach:0.95 | 1.5d test-retest |  |
| Continued. Table 2. | | | | | | | |  |
| Dominguez-Olivan P et al.  2020 [44]  Spain  ABMH and JALP  (4 March-19 April 2024) | N=30  Age (mean/SD):73.3/6.2  Gender (% male): 53.33  Disease: No  Disorder history (mean/SD):NA | BESTest:90.685/6.52  Mini-BESTest:?/? | Institutionalized normal | BESTest:Yes  Mini-BESTest: Yes  Other scale:No | Number:2 inter-rater  Profession: Physical Therapist  Experience (Yes or No/time):Yes/? | BESTest  ICC inter-rater:0.97  α de Cronbach:0.79  Mini-BESTest  ICC inter-rater:0.79  α de Cronbach:0.79 | 1 w |  |
| Alyousef NI et al.  2023 [45]  Saudi Arabia  ABMH and JALP  (4 March-19 April 2024) | N=56  Age (mean/SD):36.11/13.11  Gender (% male): 32.1  Disease: stroke, spinal cord injury, traumatic brain injury.  Disorder history (mean/SD):?/? | Mini-BESTest:20.0/5.4 | Clinical | Mini-BESTests: Yes  Other scale:No | Number: 1 intra-rater  Profession:?  Experience (Yes or No/time):Yes/? | ICC intra-rater:0.95  α de Cronbach: 0.96 | 0d intra-rater |  |
| Alqathani BA et al.  2022 [46]  Saudi Arabia  ABMH and JALP  (4 March-19 April 2024) | N=140  Age (mean/SD):66.2/6.2  Gender (% male): 45.7  Disease: No  Disorder history (mean/SD):NA | Mini-BESTest:22.79/4.7 | Institutionalized  Normal | Mini-BESTest: Yes  Other scale: No | Number: 2 inter-rater, 1 intra-rater  Profession: Physical therapist  Previous training: ?  Experience (Yes or No/time):?/? | ICC inter-rater:0.93  ICC intra-rater:0.99  α de Cronbach: 0.93 | 10d intra-rater |  |
| Franchignoni F et al.  2015 [48]  Italy  ABMH and JALP  (4 March-19 April 2024) | N=234  Age (mean/SD):65.9/13.8  Gender (% male): 44.87  Disease: stroke, Parkinson disease, sensoriomotor polyneuropathy, cerebellar ataxia, vestibular disorders, multiple sclerosis, diffuse encephalopathy, traumatic brain injury, neuromuscular disease, central nervous system | Mini-BESTest:?/? | Clinical | Mini-BESTest: Yes  Other scale:No | Number: NA  Profession: Physical Therapist  Experience (Yes or No/time):Yes/? | α de Cronbach:0.94 | NA |  |
| Continued. Table 2. | | | | | | | |  |
|  | neoplasm, hereditary ataxia, motoneuron disease.  Disorder history (mean/SD):?/? |  |  |  |  |  |  |  |
| Franchignoni F et al.  2022 [49]  Italy  ABMH and JALP  (4 March-19 April 2024) | N=193  Age (mean/SD):70/10.6  Gender (% male): 45.6  Disease: Parkinson disease  Disorder history (mean/SD):8.7/5.3 | Mini-BESTest:15.4/7.2 | Clinical | Mini-BESTest: Yes  Other scale:No | Number:NA  Profession: ?  Experience (Yes or No/time):?/? | α de Cronbach:0.94 | NA |  |
| Godi M et al.  2019 [50]  Italy  ABMH and JALP  (4 March-19 April 2024) | N=416  Age (mean/SD):66.5/14.2  Gender (% male): 45  Disease: Parkinson disease, stroke, sensoriomotor polyneuropathy, cerebellar ataxia, diffuse encephalopathy, multiple sclerosis, traumatic brain injury, vestibular disorders, | Mini-BESTest:?/?  Brief-BESTest:8.9/6.4 | Clinical | Mini-BESTest: Yes  Brief-BESTest:Yes  Other scale: No | Number: NA  Profession:?  Experience (Yes or No/time):?/? | Mini-BESTest  α de Cronbach:0.89  Brief-BESTest  α de Cronbach:0.92  Coeficiente omega: 0.75 | NA |  |
|  | neuromuscular disorders, central nervous system neoplasm.  Disorder history (mean/SD):?/? |  |  |  |  |  |  |  |
| Bravini E et al.  2016 [51]  Italy  ABMH and JALP  (4 March-19 April 2024) | N=244  Age (mean/SD): 65.3/14.9  Gender (% male): 45.49  Disease: stroke, Parkinson disease, cerebellar ataxia, sensoriomotor polyneuropathy, multiple sclerosis, vestibular disorders, diffuse encephalopathy, central nervous system neoplasm, traumatic brain injury, myopathy  Disorder history (mean/SD):?/? | Brief-BESTest:?/? | Clinical | Brief-BESTest: Yes  Other scale: No | Number: 3 inter-rater, 1 test-retest  Profession: Physical therapist  Experience: (Yes or No/time):Yes/10y | ICC inter-rater:0.90  ICC test-retest:0.94  α de Cronbach:0.89 | 1d test-retest |  |
| Continued. Table 2. | | | | | | | |  |
| Mitchell KD et al.  2018 [70]  USA  ABMH and JALP  (4 March-19 April 2024) | N=20  Age (mean/SD): 43.3/10.2  Gender (% male): 80  Disease: multiple sclerosis  Disorder history (mean/SD): ?/? | BESTest:71.1/20.8 | Clinical | BESTest: Yes  Other scale: No | Number: 1 intra-rater  Profession: physical therapist  Experience (Yes or No/ time):?/? | ICC intra-rater:0.98 | 10.5d intra-rater |  |
| Potter K et al.  2018 [71]  USA  ABMH and JALP  (4 March-19 April 2024) | N=21  Age (mean/SD): 55.9/9.6  Gender (% male): 76.19  Disease: multiple sclerosis  Disorder history (mean/SD): 13.38y/9.6 | BESTest:89.52/13.77  Mini-BESTest:?/? | Clinical | BESTest: Yes  Other scale: No | Number: 1 test-retest  Profession: physical therapist  Experience (Yes or No/ time):?/? | BESTest  ICC test-retest:0.94  α de Cronbach:0.97  Mini-BESTest  ICC test-retest:0.98 | 7d intra-rater |  |
| Wang-Hsu E et al.  2018 [72]  USA  ABMH and JALP  (4 March-19 April 2024) | Sample a (Wang-Hsu et al, 2017a)  N=32  Age (mean/SD): 85.5/5.6  Gender (% male): 35  Disease: No  Disorder history (mean/SD):NA | BESTest:67.8/8.7 | Community normal | BESTest: Yes  Other scale: No | Number: 2 inter-rater  Profession: Physical therapist, doctor  Experience (Yes or No/ time):Yes/?17y | BESTest  ICC inter-rater:0.97 | NA |  |
|  | Sample b (Wang-Hsu et al, 2017b)  N=70  Age (mean/SD): 85.0/5.5  Gender (% male): 61.43  Disease: No  Disorder history (mean/SD): NA | BESTest:68.3/9.9 | Clinical | BESTest: Yes  Other scale: No | Number: 1 test-retest  Profession: Physical therapist  Experience (Yes or No/ time):Yes/17y | BESTest  ICC test-retest:0.93 | 10.5d intra-rater |  |
| Continued. Table 2. | | | | | | | |  |
| Leddy AL et al.  2011a [73]  USA  ABMH and JALP  (4 March-19 April 2024) | N=80  Age (mean/SD):68.2/9.3  Gender (% male): 41  Disease: Parkinson disease  Disorder history (mean/SD):8.5/5.4 | BESTest:?/?  Mini-BESTest:20.2/7.0 | Clinical | BESTest: Yes  Mini-BESTest:Yes  Other scale: No | Number: 3 inter-rater; 1 intra-rater  Profession: physical therapist  Experience (Yes or No/time):Yes/12y | BESTest  ICC inter-rater.0.96  ICC intra-rater:0.88  Mini-BESTest  ICC inter-rater:0.91  ICC intra-rater:0.92 | 15d intra-rater |  |
| Leddy AL et al.  2011b [74]  USA  ABMH and JALP  (4 March-19 April 2024) | N=80  Age (mean/SD):68.2/9.3  Gender (% male): 41  Disease: Parkinson disease  Disorder history (mean/SD):8.5/5.4 | BESTest:?/?  Mini-BESTest:20.2/7.0 | Clinical | BESTest: Yes  Mini-BESTest:Yes  Other scale: No | Number: 3 inter-rater; 1 intra-rater  Profession: physical therapist  Experience (Yes or No/time):Yes/12y | BESTest  ICC inter-rater.0.96  ICC intra-rater:0.88 | 15d intra-rater |  |
| Löfgren N et al.  2014 [75]  Sweden  ABMH and JALP  (4 March-19 April 2024) | N=27  Age (mean/SD):73.0/4.0  Gender (% male): 33.33  Disease: Parkinson disease  Disorder history (mean/SD):6.2y/4 | Mini-BESTest:? | Clinical | Mini-BESTest:Yes  Other scale: No | Number: 2 inter-rater; 1 intra-rater  Profession: Physical therapist  Experience (Yes or No/time):Yes/? | α de Cronbach:0.87  ICC inter-rater:0.72  ICC test-retest:0.80 | 7d intra-rater |  |
| Molhemi F et al.  2022 [76]  Iran  ABMH and JALP  (4 March-19 April 2024) | N=32  Age (mean/SD):37.0/9.6  Gender (% male): 74  Disease: multiple sclerosis  Disorder history (mean/SD):8.8y/5.7 | Mini-BESTest:19.1/4.9 | Clinical | Mini-BESTest:Yes  Other scale: No | Number: 1 intra-rater  Profession: physical therapist  Experience (Yes or No/time):Yes/6y | α de Cronbach:0.80  ICC inter-rater:0.90 | 7d intra-rater |  |
| Continued. Table 2. | | | | | | | |  |
| Oyama Ch et al.  2018 [77]  Japan  ABMH and JALP  (4 March-19 April 2024) | N=18  Age (mean/SD): 59.9/27.0  Gender (% male): 77.77  Disease: stroke  Disorder history (mean/SD): ? | Mini-BESTest:20.0/4.2 | Clinical | Mini-BESTest:Yes  Other scale: No | Number: 4 inter-rater  Profession: physical therapist  Experience (Yes or No/time):?/? | α de Cronbach:0.87  ICC inter-rater:0.90 | 2d inter-rater |  |
| Padgett PK et al.  2012 [78]  USA  ABMH and JALP  (4 March-19 April 2024) | Sample a (Padgett PK et al, 2012a)  N=20  Age (mean/SD): 63.6/8.07  Gender (% male): 40  Disease: stroke; Parkinson disease; essential tremor; multiple sclerosis; peripheral neuropathy secondary to diabetes.  Disorder history (mean/SD):? | BESTest:?/?  Mini-BESTest:?  Brief-BESTest:?/? | Clinical/  community normal | BESTest:Yes  Mini-BESTest:Yes  Brief-BESTest:Yes  Other scale: No | Number: 3 inter-rater  Profession: physical therapist  Experience (Yes or No/time): Yes/? | BESTest  ICC inter-rater:0.985  Mini-BESTest  α de Cronbach:0.949  ICC inter-rater:0.994  Brief-BESTest  α de Cronbach:0.917  ICC inter-rater:0.994 | NA |  |
|  | Sample b (Padgett PK et al, 2012b)  N=26  Age (mean/SD): 50.0/?  Gender (% male): 61.54  Disease: stroke; Parkinson disease; essential tremor; multiple sclerosis; peripheral neuropathy secondary to diabetes.  Disorder history (mean/SD):? | Brief-BESTest:?/? | Clinical/  community normal | Brief-BESTest:Yes  Other scale: No | Number: NA  Profession: Physical therapist  Experience (Yes or No/time): ?/? | Brief-BESTest  α de Cronbach:0.856 | NA |  |
| Ross E et al.  2016 [79]  Ireland  ABMH and JALP  (4 March-19 April 2024) | N=52  Age (mean/SD): 45.73/5.65  Gender (% male): 71.15  Disease: multiple sclerosis Disorder history (mean/SD): 10.87y/2.48 | Mini-BESTest:18.95/5.88 | Clinical | Mini-BESTest: Yes  Other scale: No | Number:2 inter-rater  Profession: Physical therapist  Experience (Yes or No/time):Yes/4y | ICC inter-rater:0.976 | ? |  |
| Continued. Table 2. | | | | | | | |  |
| Roy A et al.  2021 [80]  Canada  ABMH and JALP  (4 March-19 April 2024) | N=23  Age (mean/SD):55.2/14.5  Gender (% male): 26.09  Disease: spinal cord injury  Disorder history (mean/SD):? | Mini-BESTest:17.5/5.9 | Clinical | Mini-BESTest: Yes  Other scale:No | Number: 2 inter-rater; 1 test-retest  Profession: Physical therapist  Experience (Yes or No/time): Yes/8y | ICC inter-rater:0.96  ICC test-retest:0.94 | 1.5d test-retest |  |
| Schlenstedt Ch et al.  2015 [81]  Germany  ABMH and JALP  (4 March-19 April 2024) | Sample a (Schlenstedt Ch et al, 2015a)  N=15  Age (mean/SD):73.1/7.8  Gender (% male): ?  Disease: Parkinson disease  Disorder history (mean/SD):7.8y/6.1 | Mini-BESTest:?/? | Clinical | Mini-BESTest: Yes  Other scale: FABS, Berg balance scale | Number: 3 inter-rater  Profession: psychology student and sport scientist  Experience (Yes or No/time):?/? | ICC inter-rater:0.99 | ? |  |
|  | Sample b (Schlenstedt Ch et al, 2015b)  N=17  Age (mean/SD):62.9/11.2  Gender (% male): ?  Disease: Parkinson disease  Disorder history (mean/SD):9.8y/4.9 | Mini-BESTest: 21.6/5.9 | Clinical | Mini-BESTest: Yes  Other scale: FABS, Berg balance scale | Number: 1intra-rater  Profession: psychology student or sport scientists  Experience (Yes or No/time):?/? | ICC test-retest:0.98 | 3d test-retest |  |
| Tsang Ch et al.  2013 [82]  China  ABMH and JALP  (4 March-19 April 2024) | N=106  Age (mean/SD):57.1/11.0  Gender (% male): 30.96  Disease: stroke  Disorder history (mean/SD):?/? | Mini-BESTest:19.0/? | Clinical | Mini-BESTest: Yes  Other scale: No | Number: 2 inter-rater; 1 intra-rater  Profession: physical therapist  Experience (Yes or No/time):Yes/10y | α de Cronbach:0.89; 0.93; 0.94  ICC inter-rater:0.96  ICC intra-rater:0.97 | 10d intra-rater |  |
| Continued. Table 2. | | | | | | | |  |
| Wagner S et al.  2023 [83]  Sweden  ABMH and JALP  (4 March-19 April 2024) | N=180  Age (mean/SD):51.6/15.9  Gender (% male):?  Disease: chronic pain  Disorder history (mean/SD):?/? | Mini-BESTest:19.5/? | Clinical | Mini-BESTest: Yes  Other scale: No | Number: NA  Profession: NA  Experience (Yes or No/time):NA | α de Cronbach:0.92 | NA |  |
| Wallin A et al.  2021 [84]  Sweden  ABMH and JALP  (4 March-19 April 2024) | Sample a (Wallin A et al, 2021a)  N=28  Age (mean/SD):45.5/9.4  Gender (% male): 68  Disease: multiple sclerosis  Disorder history (mean/SD):11.5/8.1 | Mini-BESTest:22.5/3.1 | Clinical | Mini-BESTest: Yes  Other scale: No | Number: 1 test-retest  Profession: Physical Therapist  Experience (Yes or No/time):?/? | ICC test-retest:0.80 | 7d |  |
|  | Sample b (Wallin A et al, 2021b)  N=26  Age (mean/SD): 53.7/8.2  Gender (% male): 73  Disease: multiple sclerosis  Disorder history (mean/SD):12.2/8.7 | Mini-BESTest:17.5/3.7 | Clinical | Mini-BESTest: Yes  Other scale: No | Number: 1 test-retest  Profession: Physical Therapist  Experience (Yes or No/time):?/? | ICC test-retest:0.83 | 7d |  |
| Winairuk T et al.  2019 [85]  Thailand  ABMH and JALP  (4 March-19 April 2024) | N=12  Age (mean/SD):58.42/13.41  Gender (% male): ?  Disease: stroke  Disorder history (mean/SD):? | Mini-BESTest:12.62/1.11  Brief-BESTest:8.32/0.53 | Clinical | Mini-BESTest:Yes  Brief-BESTest:Yes  Other scale: No | Number: 5 inter-rater, 5 intra-rater  Profession: Physical therapist  Experience (Yes or No/time):Yes/10y | Mini-BESTest  ICC inter-rater:0.95  ICC intra-rater:0.98  Brief-BESTest  ICC inter-rater:0.98  ICC intra-rater:0.98 | 7d intra-rater |  |
| Continued. Table 2. | | | | | | | |  |
| Anson E et al.  2019 [86]  USA  ABMH and JALP  (4 March-19 April 2024) | Sample a (Anson E et al, 2019a)  N=58  Age (mean/SD):78.1/7.01  Gender (% male): 72  Disease: No  Disorder history (mean/SD):NA | BESTest:72.51/8.74 | Community normal | BESTest:Yes  Other scale:No | Number: 1 test-retest  Profession:?  Experience (Yes or No/time):Yes/? | ICC test-retest: 0.86 | 32d test-retest |  |
|  | Sample b (Anson E et al, 2019b)  N=56  Age (mean/SD):78.1/7.01  Gender (% male): 72  Disease: No  Disorder history (mean/SD):NA | Mini-BESTest:19.15/3.47 | Community normal | Mini-BESTest  Other scale:No | Number: 1 test-retest  Profession:?  Experience (Yes or No/time):Yes/? | ICC test-retest: 0.84 | 32d test-retest |  |
| Bustamante-Contreras C et al.  2020 [87]  Chile  ABMH and JALP  (4 March-19 April 2024) | N=50  Age (mean/SD):69.14/8.65  Gender (% male): 38  Disease: Parkinson disease  Disorder history (mean/SD):8.17/6.01 | Mini-BESTest:?/? | Clinical | Mini-BESTest: Yes  Other scale: No | Number: 3 inter-rater  Profession:?  Experience: (Yes or No/time):Yes/? | ICC inter-rater:0.97  α de Cronbach:0.845 | NA |  |
| Cramer E et al.  2020 [88]  Germany  ABMH and JALP  (4 March-19 April 2024) | N=50  Age (mean/SD):64.58/13.34  Gender (% male): 32  Disease: stroke  Disorder history (mean/SD):? | Mini-BESTest:17.24/6.71 | Clinical | Mini-BESTest: Yes  Other scale:No | Number: NA  Profession:NA  Experience: (Yes or No/time):NA | α de Cronbach:0.9 | NA |  |
| Continued. Table 2. | | | | | | | |  |
| Godi M et al.  2013 [89]  Italy  ABMH and JALP  (4 March-19 April 2024) | Sample a (Godi M et al, 2013a)  N=93  Age (mean/SD):66.2/13.2  Gender (% male): 56.99  Disease: hemiparesis, multiple sclerosis, vestibuar disorders,  neuromuscular diseases, hereditary ataxia, sensoriomotor polyneuropathy, central nervous  system neoplasm, unspecifc age-related balance disorders.  Disorder history (mean/SD):?/? | Mini-BESTest:12.8/6.9 | Clinical | Mini-BESTest: Yes  Other scale: No | Number:NA  Profession: ?  Experience (Yes or No/time):?/? | α de Cronbach:0.90 | NA |  |
|  | Sample b (Godi M et al, 2013b)  N=32  Age (mean/SD):67.3/13.5  Gender (% male): 59.38  Disease: hemiparesis, multiple sclerosis, vestibuar disorders,  neuromuscular diseases, hereditary ataxia, sensoriomotor polyneuropathy, central nervous  system neoplasm, unspecifc age-related balance disorders.  Disorder history (mean/SD):?/? | Mini-BESTest:11.1/7.6 | Clinical | Mini-BESTest: Yes  Other scale: No | Number:3 inter-rater, 1 intra-rater  Profession: ?  Experience (Yes or No/time):?/? | ICC inter-rater:0.98  ICC intra-rater:0.96 |  |  |
| Jorgensen V et al.  2017 [90]  Sweden  ABMH and JALP  (4 March-19 April 2024) | N=46  Age (mean/SD): 54.5/17.0  Gender (% male): 30.43  Disease: spinal cord injury  Disorder history (mean/SD):?/? | Mini-BESTest:?/? | Clinical | Mini-BESTest:Yes  Other scale: No | Number: NA  Profession: ?  Experience: (Yes or No/time):?/? | α de Cronbach:0.95 | NA |  |
| Continued. Table 2. | | | | | | | |  |
| Huang M et al.  2017 [91]  China  ABMH and JALP  (4 March-19 April 2024) | N=50  Age (mean/SD): 59.2/7.3  Gender (% male):36  Disease: stroke  Disorder history (mean/SD):?/? | Brief-BESTest:12.1/5.2 | Clinical | Brief-BESTest: Yes  Other scale: No | Number: 2 inter-rater, 1 intra-rater  Profession: Physical therapist  Experience: (Yes or No/time):?/? | ICC inter-rater:0.974  ICC intra-rater:0.972  α de Cronbach:0.818 | 15 minutes intra-rater |  |
|  |  |  |  |  |  |  |  |  |
| Leung RWM et al.  2018 [92]  Australia  ABMH and JALP  (4 March-19 April 2024) | N=30  Age (mean/SD): 72.0/7.0  Gender (% male):43.3  Disease: chronic obstructive pulmonary disease  Disorder history (mean/SD):?/? | Brief-BESTest:20.2/2.6 | Clinical | Brief-BESTest:Yes  Other scale: No | Number: 2 inter-rater, 1 intra-rater  Profession: Physical therapist  Experience: (Yes or No/time):Yes/? | ICC inter-rater:0.86  ICC intra-rater:0.93 | 7d intra-rater |  |
| Chan K et al. [93]  2019  Canada  ABMH and JALP  (4 March-19 April 2024) | N=21  Age (mean/SD):56.4/14.8  Gender (% male): 66.67  Disease: spinal cord injury  Disorder history (mean/SD):7.3/9.0 | Mini-BESTest:14.0/8.7 | Clinical | Mini-BESTest: Yes  Other scale: No | Number:1 test-retest  Profession: physical therapist  Experience (Yes or No/time):?/? | ICC test-retest:0.98 | ? test-retest |  |
| Levin I et al.  2019 [94]  USA  ABMH and JALP  (4 March-19 April 2024) | N=20  Age (mean/SD): 32.7/9.3  Gender (% male):25%  Disease: cerebral palsy  Disorder history (mean/SD):?/? | BESTest:67.4/17.6 | Clinical | BESTest:Yes  Other scale: FFST, ABC, MFES, FGS. | Number: 1 test-retest  Profession: Physical therapist  Experience: (Yes or No/time):Yes/20y. | ICC test-retest: 0.99 | 10d test-retest |  |
| Continued. Table 2. | | | | | | | |  |
| Gylfadottir S et al. [95]  2023  Iceland  ABMH and JALP  (4 March-19 April 2024 | N=30  Age (mean/SD):56.6/12.1  Gender (% male): 46.7  Disease: patients attending the various inpatients interdisciplinary rehabilitation options (neurological, chronic pain, cardiac, pulmonary, vocational, obesity, psychiatry and arthritis rehabilitation).  Disorder history (mean/SD):?/? | Mini-BESTest:19.3/4.7 | Clinical | Mini-BESTest: Yes  Other scale: No | Number: 2 inter-rater, 1 intra-rater.  Profession: physical therapist students.  Experience (Yes or No/time):?/? | ICC test-retest: 0.84  ICC inter-rater:0.96 | 1-3d test-retest |  |

Abbreviations: ABC: Activities-specific Balance Confidence Scale; ABMH: Ana-Belén Meseguer-Henarejos; d:days; FGS: Fast gait speed; FFST: Four Square Step Test; JJLG: José-Antonio López-Pina; N: individuals number; ICC: intraclass correlation coefficient; MFES: Modified Falls Efficacy Scale; SD: Standard Deviation; y:years.
