# Supplementary material for: The balance evaluation systems test (BESTest), mini-BESTest and brief-BESTest as clinical tools to assess balance control across different populations: A reliability generalization meta-analysis
Source: PLoS One. 2025 Apr 3;20(4):e0318302. doi: 10.1371/journal.pone.0318302 (PMC11967966; doi:10.1371/journal.pone.0318302)
Supplement: S5 Table — (DOCX) [file pone.0318302.s005.docx]

**S5 Table. Evaluating methodological quality**

| **Study**  **Year** | **BESTest, Mini-BESTest and/or Brief-BESTest** | **Internal Consistency**  **(Box 4)** | **COSMIN Score/Quality Score** | **Reliability (Box 6)** | | | **COSMIN Score/Quality Score** |
| --- | --- | --- | --- | --- | --- | --- | --- |
|  |  |  |  | **Intra-rater** | **Test-retest** | **Inter-rater** |  |
| Horak FB et al. 2009 [1] | BESTest | NR | NR | NR | NR | ICC=0.91 | Doubtful/+ |
| Marques A et al. 2016 [13] | BESTest  Mini-BESTest  Brief-BESTest | NR  NR  NR | NR  NR  NR | NR  NR  NR | ICC=0.77  ICC=0.73  ICC=0.82 | ICC=0.86  ICC=0.71  ICC=0.93 | Doubtful/+  Doubtful/+  Doubtful/+ |
| Nakhostin-Ansari A et al. 2022 [15] | Mini-BESTest  Brief-BESTest | NR  NR | NR  NR | ICC=0.965  ICC=0.973 | NR | ICC=0.965  ICC=0.965 | Doubtful/+  Doubtful/+ |
| Wallén MB et al. 2016 [17] | Mini-BESTest | Cronbach α=0.738 | Inadequate/+ | NR | NR | NR | NR |
| Godi M et al. 2021 [18] | Mini-BESTest | ω=0.69-0.90 | Very good/+ | NR | NR | NR | NR |
| Yingyongyudha A et al. 2016 [19] | BESTest  Mini-BESTest | NR  NR | NR  NR | ICC=0.85  ICC=0.90 | NR  NR | NR  NR | Doubtful/+  Doubtful/+ |
| Phyu SN et al. 2022a [20] | Mini-BESTest | Cronbach α=0.73 | Very good/+ | ICC=0.93 | NR | ICC=0.95 | Doubtful/+ |
| Phyu SN et al. 2022b [21] | Mini-BESTest | NR | NR | NR | ICC=0.80 | NR | Doubtful/+ |
| Chinsongkram B et al. 2014 [22] | BESTest | NR | NR | ICC=0.99 | NR | ICC=0.99 | Doubtful/+ |
| Madsalaea T et al. 2022 [23] | BESTest | NR | NR | ICC=0.96 | NR | NR | Doubtful/+ |
| Rodrigues LC et al. 2014 [24] | BESTest | NR | NR | NR | ICC=0.98 | ICC=0.93 | Doubtful/+ |
| Table S2. Continued | | | | | | | |
| Pereira Viveiro LA et al. 2019 [25] | BESTest  Mini-BESTest  Brief-BESTest | NR  NR  NR | NR  NR  NR | NR  NR  NR | ICC=0.939  ICC=0.933  ICC=0.939 | ICC=0.993  ICC=0.992  ICC=0.993 | Doubtful/+  Doubtful/+  Doubtful/+ |
| Maia AC et al. 2013 [26] | BESTest  Mini-25]BESTest | NR  NR | NR  NR | Sample a  ICC=0.92  Sample b  ICC=0.98  Sample a  ICC=0.95  Sample b  ICC=0.99 | NR  NR  NR  NR | NR  NR  NR  NR | Doubtful/+  Doubtful/+  Doubtful/+  Doubtful/+ |
| Jácome C et al. 2016 [27] | BESTest  Mini-BESTest  Brief-BESTest | NR  NR  NR | NR  NR  NR | ICC=0.87  ICC=0.88  ICC=0.82 | NR  NR  NR | ICC=0.85  ICC=0.85  ICC=0.97 | Doubtful/+  Doubtful/+  Doubtful/+ |
| Jácome C et al. 2018 [28] | BESTest  Mini-BESTest  Brief-BESTest | NR  NR  NR | NR  NR  NR | NR  NR  NR | ICC=0.94  ICC=0.84  ICC=0.84 | NR  NR  NR | Doubtful/+  Doubtful/+  Doubtful/+ |
| Naghdi S et al. 2020 [29] | Mini-BESTest | NR | NR | NR | NR | ρ=0.98 | Adequate/+ |
| Bahirei S et al. 2023 [30] | BESTest | NR | NR | NR | NR | ICC=0.98 | Doubtful/+ |
| Table S2. Continued | | | | | | | |
| Lemay J-F et al. 2019 [31] | Mini-BESTest | NR | NR | ICC=0.988 | NR | ICC=0.974 | Doubtful/+ |
| Severijns P et al. 2019 [32] | BESTest | Sample b  Cronbach α=0.70 | Very good/+ | NR | NR | Sample a  ICC=0.94  Sample b  ICC=0.90 | Doubtful/+  Doubtful/+ |
| Lampropoulou SI et al. 2019 [33] | Mini-BESTest | Cronbach α=0.942 | Very good/+ | NR | ICC=0.966 | ICC=0.998 | Adequate/+ |
| Kondo Y et al. 2020 [34] | BESTest  Mini-BESTest  Brief-BESTest | NR  NR  NR | NR  NR  NR | NR  NR  NR | ICC=0.92  ICC=0.91  ICC=0.81 | NR  NR  NR | Doubtful/+  Doubtful/+  Doubtful/+ |
| Hamre Ch et al. 2017 [35] | BESTest  Mini-BESTest | NR  NR | NR  NR | NR  NR | ICC=0.79  ICC=0.87 | ICC=0.98  ICC=0.95 | Very good/+  Very good/+ |
| Goljar N et al. 2017 [36] | Mini-BESTest | Cronbach α=0.96 | Very good/+ | NR | NR | NR | NR |
| Aydogan Arslan SA et al. 2021 [37] | Brief-BESTest | NR | NR | ICC=0.965 | NR | ICC=0.95 | Doubtful/+ |
| Göktas A et al. 2020 [38] | Mini-BESTest | Cronbach α=0.967 | Very good/+ | NR | ICC=0.994 | NR | Doubtful/+ |
| Dogrouz Karatekin B et al.  2023 [39] | Mini-BESTest | NR | NR | NR | ICC=0.998 | ICC=0.989 | Doubtful/+ |
| Table S2. Continued | | | | | | | |
| Dewar R et al. 2017 [40] | BESTest  Mini-BESTest | NR  NR | NR  NR | NR  NR | Sample a  ICC=0.96  Sample b  ICC=0.83  Sample a  ICC=0.86 | Sample a  ICC=0.87  NR  Sample a  ICC=0.56 | Doubtful/+  Doubtful/+  Doubtful/± |
| Huang MH et al. 2016 [41] | BESTest  Mini-BESTest  Brief-BESTest | NR  NR  NR | NR  NR  NR | NR  NR  NR | ICC=0.92  ICC=0.90  ICC=0.94 | ICC=0.96  ICC=0.86  ICC=0.92 | Doubtful/+  Doubtful/+  Doubtful/+ |
| Chan ACM et al. 2015 [42] | BESTest  Mini-BESTest  Brief-BESTest | Sample a  Cronbach α=0.98  Sample b  Cronbach α=0.96  Sample b  Cronbach α=0.97 | Very good/+  Very good/+  Very good/+ | Sample a  ICC=0.96  NA  Sample b  ICC=0.94 | NA  NA  NA | Sample a  ICC=0.99  Samples a,b  ICC=0.96  Sample a  ICC=0.97 | Doubtful/+  Doubtful/+  Doubtful/+ |
| Table S2. Continued | | | | | | | |
| Chiu AYY et al. 2018 [43] | BESTest  Mini-BESTest  Brief-BESTest | NR  Rater A  Subscale  1: α=0.90  2: α=0.98  3: α=0.81  4: α=0.97  Rater B  Subscale  1: α=0.80  2: α=0.97  3: α=0.85  4: α=0.95  Cronbach α=0.95 | NR  Very good/+  Very good/+ | NR  NR  NR | ICC=0.99  ICC=0.80  ICC=0.99 | ICC=0.99  ICC=0.81  ICC=0.97 | Doubtful/+  Doubtful/+  Doubtful/+ |
| Dominguez-Olivan et al. 2020 [44] | BESTest  Mini-BESTest | Cronbach α=0.79  Cronbach α=0.79 | Very good/+  Very good/+ | NR  NR | NR  NR | ICC=0.97  ICC=0.79 | Doubtful/+  Doubtful/+ |
| Alyousef NI et al. 2023 [45] | Mini-BESTest | Cronbach α=0.96 | Very good/+ | NR | ICC=0.95 | NR | Doubtful/+ |
| Alqathani BA et al. 2022 [46] | Mini-BESTest | Cronbach α=0.93 | Very good/+ | ICC=0.99 | NR | ICC=0.93 | Doubtful/+ |
| Franchignoni F et al. 2015 [47] | Mini-BESTest | Cronbach α=0.94 | Very good/+ | NR | NR | NR | NR |
| Franchignoni F et al. 2022 [48] | Mini-BESTest | Cronbach α=0.94 | Very good/+ | NR | NR | NR | NR |
| Table S2. Continued | | | | | | | |
| Godi M et al. 2019 [49] | Mini-BESTest    Brief-BESTest | Cronbach α=0.89  Cronbach α=0.92 | Very good/+  Very good/+ | NR | NR | NR | NR |
| Bravini E et al. 2016 [50] | Brief-BESTest | Cronbach α=0.89 | Very good/+ | NR | ICC=0.94 | ICCC=0.90 | Doubtful/+ |
| Mitchell KD et al. 2018 [70] | BESTest | NR | NR | ICC=0.98 | NR | NR | Doubtful/+ |
| Potter K et al. 2019 [71] | BESTest  Mini-BESTest | Cronbach α=0.97  NR | Very good/+  NR | NR  NR | ICC=0.97  NR | NR  ICC=0.98 | Doubtful/+  Doubtful/+ |
| Wang-Hsu E et al. 20118 [72] | Sample a  BESTest  Sample b  BESTest | NR  NR | NR  NR | NR  NR | NR  ICC=0.93 | ICC=0.97  NR | Doubtful/+  Doubtful/+ |
| Leddy A et al. 2011a [73] | BESTest  Mini-BESTest | NR  NR | NR  NR | ICC=0.88  ICC=0.92 | NR  NR | ICC=0.96  ICC=0.91 | Doubtful/+  Doubtful/+ |
| Leddy A et al. 2011b [74] | BESTest | NR | NR | ICC=0.88 | NR | ICC=0.96 | Doubtful/+ |
| Löfgren N et al. 2014 [75] | Mini-BESTest | Cronbach α=0.87 | Very good/+ | NA | ICC=0.80 | ICC=0.72 | Doubtful/+ |
| Molhemi F et al. 2022 [76] | Mini-BESTest | Cronbach α=0.80 | Very good/+ | NR | NR | ICC=0.90 | Very good/+ |
| Oyama Ch et al. 2018 [77] | Mini-BESTest | Cronbach α=0.87 | Very good/+ | NR | NR | ICC=0.90 | Doubtful/+ |
| Table S2. Continued | | | | | | | |
| Padgett PK et al. 2012 [78] | Sample a  BESTest  Mini-BESTest  Brief-BESTest  Sample b  Brief-BESTest | NR  Cronbach α=0.949  Cronbach α=0.917  Cronbach α=0.856 | NR  Very good/+  Very good/+  Very good/+ | NR  NR  NR  NR | NR  NR  NR  NR | ICC=0.985  ICC=0.994  ICC=0.994  NR | Doubtful/+  Doubtful/+  Doubtful/+  NR |
| Ross E et al. 2016 [79] | Mini-BESTest | NR | NR | NR | NR | ICC=0.976 | Doubtful/+ |
| Roy A et al. 2021 [80] | Mini-BESTest | NR | NR | NR | ICC=0.94 | ICC=0.96 | Adequate/+ |
| Schlendstedt C et al. 2015 [81] | Mini-BESTest | NR | NR | NR | Sample a  NR  Sample b  ICC=0.98 | Sample a  ICC=0.99  Sample b  NR | Very good/+  Very good/+ |
| Tsang Ch et al. 2013 [82] | Mini-BESTest | Rater 1  Cronbach α=0.89  Rater 2  Cronbach α=0.93  Rater 3  Cronbach α=0.94 | Very good/+ | ICC=0.97 | NR | ICC=0.96 | Doubtful/+ |
| Wagner S et al. 2023 [83] | Mini-BESTest | Cronbach α=0.92 | Very good/+ | NR | NR | NR | NR |
| Table S2. Continued | | | | | | | |
| Wallin A et al. 2021 [84] | Mini-BESTest | NR | NR | NR | Sample a  ICC=0.80  Sample b  ICC=0.83 | NR  NR | Doubtful/+  Doubtful/+ |
| Winairuk T et al. 2019 [85] | Mini-BESTest  Brief-BESTest | NR  NR | NR  NR | ICC=0.98  ICC=0.98 | NR  NR | ICC=0.95  ICC=0.98 | Doubtful/+  Doubtful/+ |
| Anson E et al. 2019 [86] | Sample a  BESTest  Sample b  Mini-BESTest | NR  NR | NR  NR | NR  NR | ICC=0.86  ICC=0.84 | NR  NR | Doubtful/+  Doubtful/+ |
| Bustamante-Contreras C et al. 2020 [87] | Mini-BESTest | Cronbach α=0.845 | Very good/+ | NR | NR | ICC=0.97 | Doubtful/+ |
| Cramer E et al. 2020 [88] | Mini-BESTest | Cronbach α=0.90 | Very good/+ | NR | NR | NR | NR |
| Godi M et al. 2013 [89] | Mini-BESTest | Sample a  Cronbach α=0.90  Sample b  NR | Very good/+  NR | NR  NR | NR  ICC=0.96 | NR  ICC=0.98 | NR  Inadequate/+ |
| Jorgensen V et al. 2017 [90] | Mini-BESTest | Cronbach α=0.95 | Very good/+ | NR | NR | NR | NR |
| Huang M et al. 2017 [91] | Brief-BESTest | Cronbach α=0.818 | Very good/+ | ICC=0.972 | NR | ICC=0.974 | Doubtful/+ |
| Leung RWM et al. 2018 [92] | Brief-BESTest | NR | NR | ICC=0.93 | NR | ICC=0.86 | Doubtful/+ |
| Table S2. Continued | | | | | | | |
| Chan K et al. 2019 [93] | Mini-BESTest | NR | NR | NR | ICC=0.98 | NR | Doubtful/+ |
| Levin I et al. 2019 [94] | BESTest | NR | NR | NR | ICC=0.99 | NR | Doubtful/+ |
| Gylfadottir S et al. 2023 [95] | Mini-BESTest | Cronbach α=0.80 | Very good/+ | ICC=0.86 | ICC=0.84 | ICC=0.96 | Very good/+ |

*Abbreviations:* GRC: Global rating of change; ICC: Intraclass correlation coefficient; MDC_95_: Minimal detectable change al 95%; MIC: Minimal important change; NR: Not reported; ρ: Pearson correlation coefficient; SEM: Standard error of measurement.
